# Measuring individual and community capacity factors in people with multimorbidity and exploring associations with health outcomes

**DOI:** 10.1186/s12916-025-04337-y

**Published:** 2025-10-16

**Authors:** Marianne McCallum, Frances S Mair, Sara Macdonald, Mary Kathleen Hannah, Kathryn Skivington, Jim Lewsey

**Affiliations:** 1 General Practice and Primary Care, School of Health and Wellbeing, University of Glasgow, Glasgow, UK; 2 MRC/CSO Social and Public Health Sciences Unit, School of Health and Wellbeing, University of Glasgow, Glasgow, UK; 3 Heath Economics and Health Technology Assessment, School Health and Wellbeing, University of Glasgow, Glasgow, UK

**Keywords:** Multimorbidity, Burden of Treatment Theory, Treatment burden, Capacity

## Abstract

**Background:**

People with multimorbidity work to manage their conditions (burden of treatment). Burden of Treatment Theory (BOTT) proposes poorer outcomes when this work outweighs capacity — an individual’s ability to successfully undertake the work of self-management. Capacity is influenced by individual and community factors. This study aims to quantify individual and community capacity factors and explore associations, if any, with mortality and hospitalisation in people with multimorbidity.

**Methods:**

Data source is as follows: West of Scotland Twenty-07 cohort (three age-based cohorts — 15, 35 and 55 years at baseline (wave 1), followed up with four additional waves over 20 years). Participants are as follows: people with ≥ 2 chronic conditions. Variables (e.g. car access/self-esteem/neighbourliness) mapped to underlying individual and community BOTT constructs. Directed acyclic graphs (DAGs) informed analysis. Cox regression analysis using time-varying covariates explored mortality associations; multiple logistic regression explored hospitalisation associations. Both analyses were adjusted for age, sex, socioeconomic deprivation (SED), alcohol, exercise, fruit/vegetable intake, BMI, smoking, marital status, number of long-term conditions and blood pressure. Exploratory analysis of potential moderating effect of SED was also undertaken.

**Results:**

A total of 2249 people had multimorbidity across the five waves (mean age 51.5 (*SD* 11.6) at baseline and 61 (14.9) at wave 5; male 40.6% baseline, 41.1% wave 5; smokers 32.7% baseline, 25.3% wave 5). Living in social housing was associated with increased mortality (*HR* (95% CI) 1.39 (1.14, 1.68)), while registered disability was associated with increased risk of hospitalisation (*OR* (95% *CI*) 1.7 (1.27, 2.27)). Feeling fearful about walking in the dark was associated with mortality (“try to avoid” OR (95% *CI*) 0.74 (0.60, 0.92); “feel uncomfortable” (OR (95% *CI*) 0.70 (0.55, 0.89); “no worries” 0.69 (0.57, 0.83)). Feeling little control over one’s life: disagreeing quite a bit with “care from others helps me to get well” OR (95% *CI*) 0.53 (0.33, 0.86). Initial exploratory analysis suggests high SED could act as a potential moderator, increasing associations between community factors with mortality and hospitalisations.

**Conclusions:**

Individual and community factors influencing capacity to self-manage multimorbidity are quantifiable and associated with adverse health outcomes. Our work adds to the growing body of evidence that capacity issues may be important when designing future multimorbidity interventions and services.

**Supplementary Information:**

The online version contains supplementary material available at 10.1186/s12916-025-04337-y.

## Background

As prevalence of chronic disease has risen, so too has the number of people living with multimorbidity (two or more chronic conditions), also sometimes termed multiple long-term conditions (MLTCs). Indeed, multimorbidity is now the norm in many high-income countries [1–3] and is increasing in prevalence in low-income countries [4, 5]. People living with multimorbidity have higher rates of premature mortality [6–8], higher healthcare utilisation [6, 9, 10] and reduced quality of life [6, 11, 12] compared to those who do not have multimorbidity. Critically, the proportion of people living with multimorbidity is socially patterned: it is more prevalent and begins at an earlier age in areas of high socioeconomic deprivation (SED) [1, 9, 13].

The volume of work required to manage multimorbidity and its impact on wellbeing is increasingly recognised as important [14, 15]. This increased workload falls predominantly on patients and their supporters [14, 16]. Burden of Treatment Theory (BOTT) proposes that where treatment burden is greater than the capacity of patients (and their support networks, including practitioners) to meet these self-care demands, then poorer outcomes will occur [14, 17]. Multimorbidity both increases treatment burden and can reduce capacity to self-manage [2, 14, 17]. Factors that may reduce capacity (e.g. reliance on public transport, lower literacy) are more common in areas experiencing high SED [18, 19].

In recent years, there has been increasing work that has demonstrated the importance of treatment burden and capacity in the contexts of chronic illness[20, 21]. BOTT, and the constructs of both treatment burden and capacity, have been applied in several contexts: stroke [22–24], heart failure [22], cancer [25] and multimorbidity in low- and middle-income countries (LMIC) [26–29]. They have also been used in a domestic violence setting [30]. This has resulted in the development of several measures [31–33] demonstrating high levels of treatment burden for people with multimorbidity [34–36], especially in the context of severe socioeconomic deprivation [36].

In contrast, capacity has been relatively under-researched with no measures developed and limited understanding of the key factors that influence it [20]. BOTT focuses on individual-level factors and splits them into two main categories: mobilising factors (the resource people have access to) and expressing factors (whether people can use the resource they have).

The impact of certain single-capacity factors on health outcomes, and healthcare utilisation, has been extensively explored in relation to single chronic conditions [37–42]. Factors such as social networks [38, 39, 41, 42], tenancy [37], health literacy [40] and transport access [37] all impact a wide range of health outcomes in people affected by different individual chronic conditions. In the context of multimorbidity, SED and low educational level are both separately associated with mortality [43], as is registered disability in older adults [44, 45]. Otherwise, work exploring the impact of individual capacity factors on mortality, or hospitalisation, in the context of multimorbidity, is limited. How a range of these factors influence outcomes, and which ones are particularly important, especially in relation to multimorbidity, remains unclear.

Furthermore, place-based (or neighbourhood) characteristics are known to influence health [46], but their impact on multimorbidity is less clear. Community experiences have been shown to influence decisions regarding self-management of multimorbidity in a high SED context [47]. A recent scoping review suggested that place factors may have an independent influence on multimorbidity, and factors beyond SED, such as greenspace and social cohesion, remain particularly under-explored [48]. Therefore, while the association of SED and mortality in the context of multimorbidity is well established [7], the impact of area-level factors on the prevalence of multimorbidity, let alone their influence on mortality and hospitalisation, is uncertain [49, 50].

To our knowledge, there is no research that has looked at how a broad range of individual capacity, or community, factors are associated with the risk of mortality or healthcare utilisation in the context of multimorbidity. Understanding the extent to which these factors influence outcomes such as mortality and hospital admissions could enable cost–benefit analyses and inform future service and intervention design in this area. Therefore, this work aimed to use existing BOTT constructs to identify and quantify individual and community capacity factors and explore the association, if any, of capacity (individual or community) factors on mortality and self-reported hospital admissions in people with multimorbidity.

## Methods

### Study design and participants

This analysis used data on participants with multimorbidity from the West of Scotland Twenty-07 (WoS 20–07) cohort (WoS 20–07 *n* = 4510, multimorbidity subset *n* = 2249). WoS 20–07 was designed to “investigate longitudinally, the social process producing or maintaining inequalities” [51]. It followed up three separate age-based cohorts (aged 15, 35 and 55 years at baseline) from 1987–1988 to 2007–2008; baseline data collection was followed by four waves of data collection over 20 years. It collected information on four broad categories, personal life circumstances, local life circumstances, beliefs/attitudes/values and behaviours, and is described in detail elsewhere [51].

Two samples were recruited: a regional sample from 52 postcodes across the West of Scotland regions and a locality sample from a further 10 postcodes which aimed to allow a closer study of the relationship between environment and health. Variables collected from both samples across the five longitudinal waves of WoS 20–07 were used.

A total of 4510 participants were recruited at baseline with four follow-up waves; this analysis used a subset who had multimorbidity at any point during the 20 years of follow-up (*n* = 2249): baseline (*n* = 648), 1990–1991 (*n* = 887), 1995–1997 (*n* = 857), 2000–2004 (*n* = 1023) and 2007–2008 (*n* = 1280). Comparison with census data showed that the regional sample of the main cohort was broadly representative of the West of Scotland (which has a higher prevalence of SED than the UK population as a whole) [52].

The previous work [53] identified participants within the cohort who had two or more health conditions, using the Royal College of General Practitioners’ 1986 classification system codes from the chronic conditions list initially compiled by Barnett et al. [1]. A subset dataset was created including anyone in the cohort with two or more chronic conditions at any wave in the cohort (*n* = 2249). Therefore, some participants’ variables were collected prior to them having multimorbidity. In addition, a small number were coded as having multimorbidity at one wave and not in a future wave. The review of these participants identified relapsing and remitting conditions (e.g. depression/anxiety, migraines), so participants may not have self-identified these conditions if they were quiescent at the data collection point. A sensitivity analysis, with the presence of multimorbidity as a time-varying covariate, showed minimal changes on effect size and confidence intervals (supplementary material).

### Capacity variables

Different methods of questionnaire delivery were used in wave 3 between the locality and regional samples, resulting in higher missingness in this wave due to a lower response rate from the postal survey. There were also differences in the data collected between the three age cohorts in the first two waves and in how some variables were measured between waves. M. M. manually looked through the questionnaire for each wave and cohort, mapping variables collected to the underlying BOTT capacity constructs where relevant [54]. She also identified community-level factors collected at each wave. These were discussed with all the authors, including experts in BOTT (F. M./S. M.) and statistics (J. L.) and those with an in-depth knowledge of the cohort and the variables collected (M. H./K. S.). This resulted in three groups of variables. Individual variables split into the existing BOTT constructs, mobilising and expressing, and community factors:

- *Individual capacity (mobilising)*: The resource people have access to (variables: income; tenancy; employment status; life events; contact with family and friend; people to share feelings with, confide in, ask for practical support; loneliness; being a carer)
- *Individual capacity (expressing)*: Factors that influence whether people can use the resources they have (variables: self-esteem, self-reported health, health for age, health locus of control, mastery, disability, life-limiting long-term condition, anxiety, depression, educational attainment, cognition, community group involvement)
- *Community factors*: Characteristics of the wider community that may enhance or diminish individual capacity (variables: feelings about area, neighbourliness, walking in the dark, neighbourhood problems)

**Fig. 1:**
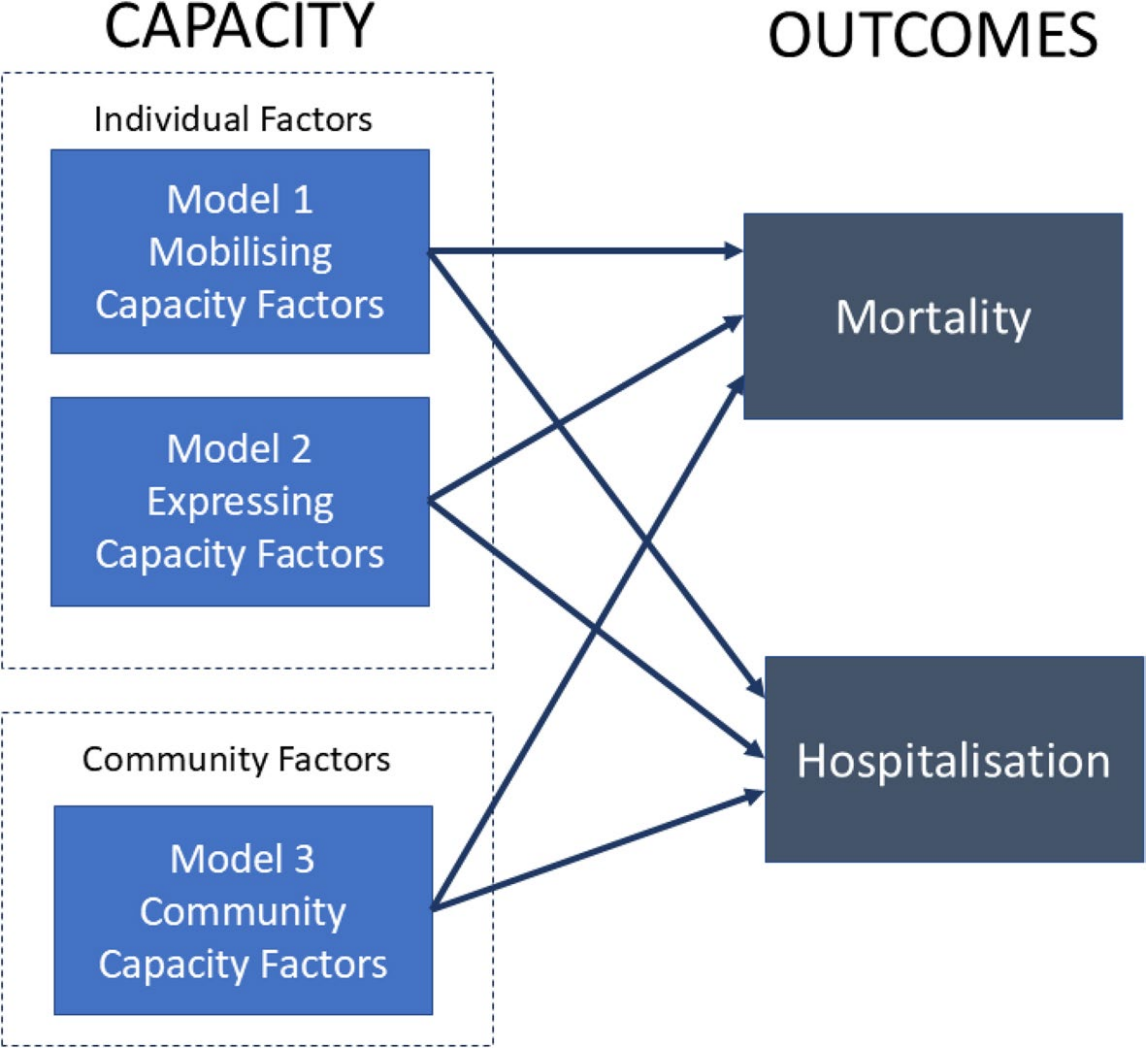
Summary of analysis

How these three groups were used within the analysis is summarised in Fig. 1.

Directed acyclic graphs (DAGs) were used to explore the relationship between the proposed variables and mortality. Potential confounder variables were then added and their relationship to both potential variables and outcomes mapped. This served two purposes: it allowed mapping of assumptions behind the analysis from the outset, and it helped determine whether potential confounders were confounders or mediators. The DAGs (available in the supplementary file) were constructed using regular discussions between some of the authors (M. M./J. L./S. M./F. M.), experts in BOTT and a patient and public involvement and engagement representative.

Socioeconomic deprivation (SED) was measured using the Carstairs score, an area-based summary measure of relative deprivation, for each participant’s postcode [55]. The Carstairs scores were used to divide the cohort into the recognised Carstairs deprivation categories (DEPCAT) from 1 (most affluent) to 7 (most deprived), based on cut-offs from the 2001 census table [55].

It was challenging to classify SED as either a confounder or mediator due to the complex relationship between it, the outcomes and many of the variables and confounders of interest. It was treated as a confounder in the main analysis, but an early exploratory analysis on an affluent and a deprived subset was also conducted on the community capacity construct to explore the relationship further.

There were several variables (e.g. self-esteem) that were likely important capacity factors but not asked in each wave. In these situations, a similar question was used as a proxy. Where this was not an option, and the variable was important, it was discussed by several authors (M. M./J. L./F. M./S. M.) before a final decision was made on inclusion. Detailed information on each of the final variables for each model, how each was measured and the degree of missingness (between waves and cohorts) are available in the supplementary information file.

### Outcome measures

All cohort participants had data linkage with the National Records of Scotland, which means that mortality data up to March 2020 was available for all participants. Our secondary outcome measure, hospitalisation, was based on self-report of hospitalisations within the preceding year and collected at each wave.

### Statistical analysis

Each pair of the potential groups of variables was examined to ensure no strong collinearity (cut-off 0.8) between them. Patterns of missingness within each of these groups of variables were reviewed and within wave and cohort missingness was managed using multivariable imputation by chained equations (MICE) under a missing at random (MAR) assumption [56]. Where data were missing due to questions not being asked to specific subsets, this is made clear in the tables summarising the variables (supplementary information) so that results can be interpreted within this context.

Unadjusted Cox regression models were constructed for mortality on both baseline data and then with time-varying covariates. Multivariable logistic regression models for self-reported hospital admission in the last year were also constructed for each of the groups. To account for repeated measurements over time in the same individual, cluster-robust standard errors were used in the regression. The DAGs for each group were used to inform selection of potential confounders for adjustment.

An exploratory analysis of the potential moderating effect of SED was undertaken by splitting the original cohort into two subsets by DEPCAT categories, as has been done previously [57]: low (DEPCAT 1–3 — more affluent) and high (DEPCAT 4–7 — more deprived). Given the paucity of work exploring place-based factors in the context of multimorbidity [48], including whether these might be moderated by SED, an initial exploratory analysis, using the DEPCAT subsets, was conducted on the community construct model.

## Results

### Participants

**Fig. 2:**
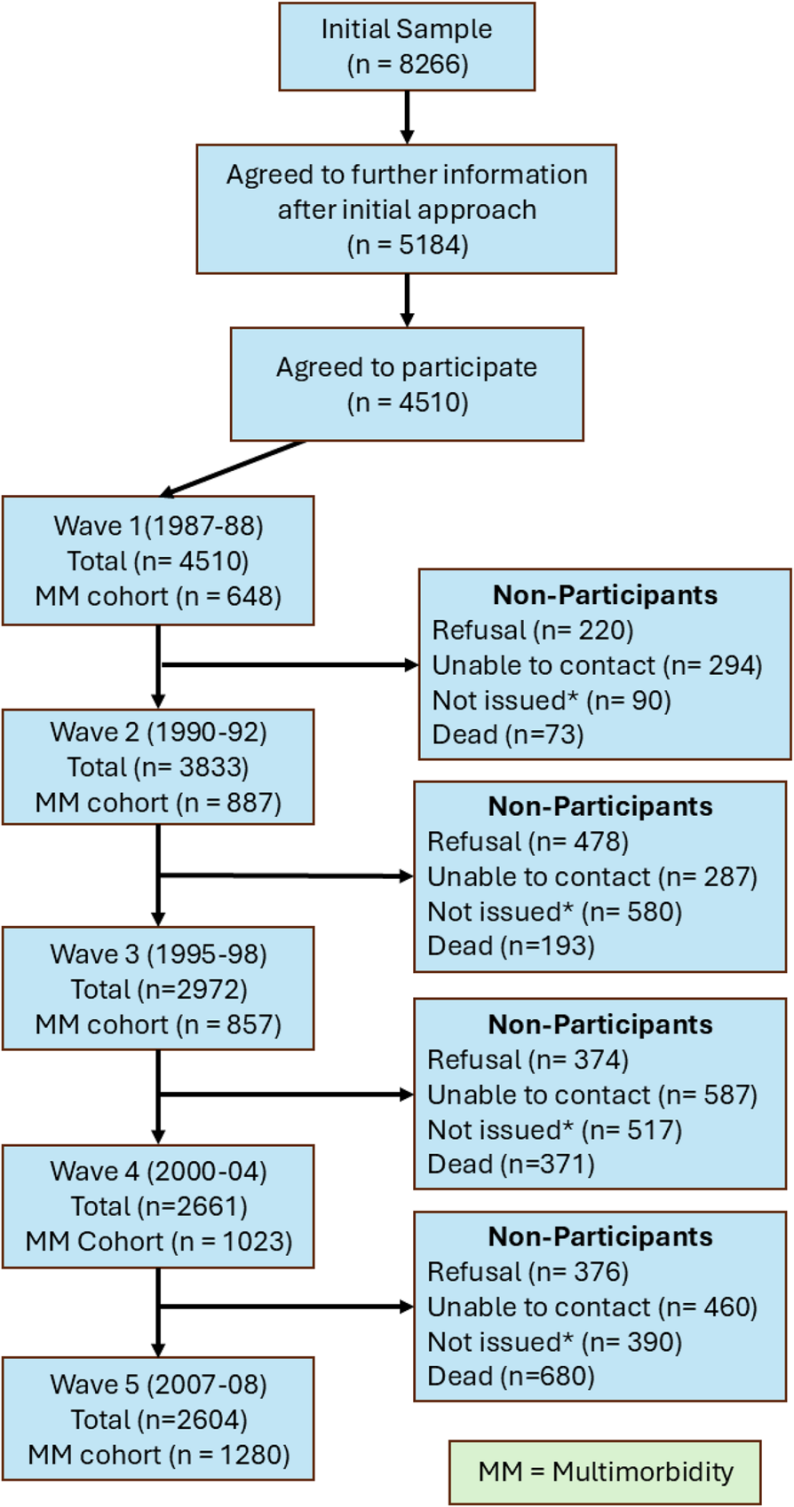
Study flow diagram summarising the numbers (total and multimorbidity (MM) subset) at each stage, as well as summarising the numbers missing and why at each stage.*In waves 2 and 3, the decision was made not to contact participants who had refused in a previous wave, moved from the areaor withdrawn from the study. From wave 4, there was a change in policy, and only those who had specifically withdrawn were not contacted

Figure 2 shows the study flow diagram showing participant numbers (total and multimorbidity), and summarising the missing data, at each wave.

Table 1 demonstrates key descriptive statistics for the multimorbidity cohort at baseline and wave 5 compared to the main cohort.

**Table 1:** Table comparing age, sex, SED, ethnicity and proportion of smokers between the main WoS20-07 cohort and the multimorbidity subset

	**Main WoS20-07 cohort at baseline (wave 1) (*n* = 4510)**	**Multimorbidity subset at baseline (W1) (*n* = 648)**	**Main WoS20-07 cohort at wave 5 (*n* = 2604)**	**Multimorbidity subset at wave 5** **(*n* = 1280)**
Age, years —				
mean (SD)	36.2 (16.7)	51.5 (11.6)	54.6 (15.4)	61.0 (14.9)
Sex (*n*)				
Male	46.5% (2095)	40.6% (263)	44.6% (1160)	41.1% (526)
Female	53.6% (2415)	59.4% (385)	55.5% (1444)	58.9% (754)
Socio-economic Status (*n*)				
DEPCAT 1 (most affluent)	5.7% (255)	4.3% (28)	3.2% (82)	3.1% (39)
DEPCAT 2	6.3% (286)	5.3% (34)	16.5% (430)	14.5% (185)
DEPCAT 3	9.9% (445)	5.9% (38)	14.8% (386)	12.7% (163)
DEPCAT 4	26.8% (1207)	23.3% (151)	23.8% (619)	22.4% (286)
DEPCAT 5	11.2% (497)	8.3% (54)	14.1% (368)	16.1% (206)
DEPCAT 6	20.3% (915)	27.8% (180)	12.5% (326)	14.0% (179)
DEPCAT 7 (most deprived)	20.1% (905)	25.2% (163)	13.9% (363)	17.0% (217)
Missing	-	-	1.1% (30)	0.4% (5)
Ethnicity (*n*)				
White	98.1% (4424)	98.8% (640)	Only recorded at baseline	
Other	1.1% (48)	0.6% (4)		
Missing	0.8% (38)	0.6% (4)		
Proportion smokers (*n*)				
Non-Smokers	48.6% (2190)	45.4% (294)	43.7% (1140)	38.2% (490)
Ex-Smokers	16.5% (743)	21.8% (141	31.7% (827)	36.2% (463)
Current Smokers	34.7% (1565)	32.7% (212)	24.0% (626)	25.3% (324)
Missing	0.3% (12)	0.2% (1)	0.4% (11)	0.2% (3)

As expected, the mean age in the multimorbidity subset was slightly older, more likely to be female, to belong to the most deprived DEPCAT, and these participants were less likely to be non-smokers. Due to the almost universal white ethnicity in the cohort, it was decided not to include ethnicity as a confounder in the analysis.

#### Individual capacity factors — mobilising capacity

There was an association between tenancy and mortality and between some employment status and both outcome measures. Of note, having fewer people to share feelings was associated with a reduced risk of death, but there was no similar association with having someone to confide in (a similar question). The association of loneliness and mortality is also unclear. Compared to those who never felt lonely, seldom feeling lonely was associated with a reduced risk of death, while those who often felt lonely had an increased risk. There was no association between loneliness and hospitalisation. Experiencing a divorce in the last year was associated with an increased risk of death, while the death of a family member showed a reduced risk.

The hazard ratios for mortality and odds ratios for hospital admission for each construct are presented in the tables below (Table 2).

**Table 2:** Relationship between mobilising capacity construct variables, mortality and hospitalisation

Variables	Hazard ratio (95% confidence interval) for mortality		Odds ratio for self-reported hospital admission in the last year^a^ (95% confidence interval)
	Baseline model^a^ (using baseline covariates)	Time-varying model^a^ (adjusting for time-varying covariates)	
Equivalised household income	1.00 (0.99, 1.00)	1.00 (1.00, 1.00)	1.00 (1, 1)
Housing tenancy			
Owner Social housing Private rent Other	1 1.06 (0.85, 1.31) 1.01 (0.49, 2.09) 1.11 (0.62, 1.98)	1 1.23 (1.10, 1.63)** 1.26 (0.71, 2.22) 1.28 (0.63, 2.65)	1 1.16 (0.85, 1.58) 1.47 (0.74, 2.93) 1.52 (0.51, 4.57)
Access to a car			
Yes No	1 1.03 (0.86, 1.25)	1 1.14 (0.95, 1.38)	1 1.00 (0.75,1.35)
Employment status			
Employed/self-employed Full-time education Carer/housewife Retired Unemployed Disabled	1 1.32 (0.40, 4.37) 1.14 (0.89, 1.46) 1.51 (1.04, 2.19)** 1.53 (1.15, 2.03)** 1.42 (1.10, 1.83)**	1 3.64 (0.82, 16.25) 1.97 (1.24, 3.15)** 1.55 (1.08, 2.22)** 1.80 (0.95, 3.39) 2.61 (1.79, 3.81)**	1 0.71 (0.07, 7.00) 2.19 (1.42, 3.39) 1.67 (0.96, 2.90) 1.21 (0.56, 2.61) 2.13 (1.33, 3.42)*
Seen family member in the last month?			
Yes No		1 1.34 (0.55, 3.27)	1 0.71 (0.39, 1.29)
Seen friend in the last month?			
Yes No		1 0.69 (0.25, 1.48)	1 1.12 (0.62, 2.02)
Number of people you can rely on for practical support			
None Up to five Five to 10 More than 10		1 1.03 (0.74, 1.41) 1.01 (0.69, 1.47) 1.06 (0.61, 1.85)	1 1.32 (0.75, 2.31) 1.44 (0.78, 2.63) 1.44 (0.63, 3.29)
Do you ever feel lonely?			
Never Seldom Occasionally Quite often Most of the time	1 0.84 (0.63, 1.20) 0.97 (0.77, 1.21) 1.20 (0.88, 1.63) 0.88 (0.55, 1.40)	1 0.68 (0.51, 0.92)** 0.88 (0.70, 1.11) 1.66 (1.21, 2.28) ** 0.90 (0.57, 1.44)	1 0.93 (0.63, 1.36) 0.70 (0.5, 0.99) 1.19 (0.75, 1.88) 1.22 (0.59, 2.52)
Someone you can share your feelings with?			
All Some feelings A few feelings	1 0.98 (0.81, 1.19) 0.85 (0.56, 1.28)	1 0.78 (0.63, 0.97)* 0.69 (0.49, 0.96)*	1 0.84 (0.61, 1.16) 0.63 (0.35, 1.14)
Someone you can confide in?			
Very frequently More often than not Occasionally Never	1 0.80 (0.57, 1.14) 1.01 (0.77, 1.32) 1.02 (0.78, 1.35)	1 1.15 (0.77, 1.73) 1.18 (0.81, 1.71) 1.05 (0.73, 1.51)	1 1.14 (0.6, 2.18) 0.80 (0.44, 1.46) 0.93 (0.52, 1.68)
Are you a carer?			
Yes No	1 0.85 (0.72, 0.99)*	1 0.72 (0.57, 0.90)**	1 0.77 (0.57, 1.03)
Divorced in the last year?			
Yes No	1 0.62 (0.23, 1.73)	1 1.96 (1.05, 3.67)*	1 1.57 (0.64, 3.84)
Become unemployed in the last year?			
Yes No	1 0.92 (0.37, 2.27)	1 0.74 (0.31, 1.78)	1 0.81 (0.25, 2.66)
Employment change in the last year?			
Yes No	1 0.81 (0.37, 1.76)	1 1.15 (0.56, 2.34)	1 0.46 (0.17, 1.23)
Death in the family			
No Yes	1 0.86 (0.57, 1.23)	1 0.74 (0.59, 0.94)*	1 0.71 (0.39, 1.29)
Death in the family			
No Yes	1 1.55 (0.80, 3.17)	1 0.84 (0.66, 1.06)	1 1.19 (0.82, 1.73)

^*^p < 0.05. **p < 0.01

^a^Adjusted for age, sex, alcohol, exercise, fruit and vegetable intake, BMI, smoking, marital status, blood pressure and number of long-term conditions. In addition, the presented results for each capacity variable are also adjusted for the other capacity variables in the mobilising group (e.g. the row for tenancy results has been adjusted for income, car access, employment status, seen family in last month, seen friend in last month, practical support, loneliness, someone to share feelings, someone to confide in and life events: divorce, unemployment, employment change and death of family or friend)

#### Individual capacity factors — expressing capacity (Table 3)

**Table 3:** Relationship between expressing capacity construct variables, mortality and hospitalisation

**Variables**	**Hazard ratio (95% confidence interval) for mortality**		**Odds ratio for ** **self-reported hospital admission in the last year** ^**a**^ **(95% confidence interval)**
	Baseline model^a^ (using baseline covariates)	Time-varying model^a^ (adjusting for time-varying covariates)	
Over the last 12 months, would you say your health on the whole has been……			
Good Fairly good Not good	1 0.85 (0.60, 1.21) 1.23 (0.65, 2.33)	1 1.12 (0.82, 1.51) 1.17 (0.76, 1.79)	1 2.76 (1.92, 3.95)** 7.47 (4.40, 12.67)**
Would you say that for someone your own age your health in general is…			
Excellent Good Fair Poor	1 1.23 (0.84, 1.80) 2.02 (1.21, 3.38)** 1.34 (0.54, 3.31)	1 0.95 (0.66, 1.36) 1.39 (0.89, 2.18) 2.09 (1.13, 3.90)**	1 0.77 (0.51, 1.15) 0.64 (0.38, 1.10) 0.74 (0.35, 1.57)
Registered disability			
No Yes	Not asked at baseline	1 1.67 (1.25, 2.23)**	1 1.91 (1.26, 2.89)**
Depression			
No Mild Moderate Severe	1 0.88 (0.57, 1.34) 0.82 (0.38, 10.77) NA	1 1.04 (0.73, 1.48) 0.72 (0.40, 1.29) 13.55 (2.3, 79.89)	1 1.08 (0.69, 1.68) 1.32 (0.69, 2.52) 2.57 (0.30, 22.3)
Anxiety			
No Mild Moderate Severe	1 0.78 (0.57, 1.07) 0.83 (0.54, 1.26) 0.59 (0.21, 1.64)	1 1.10 (0.82, 1.48) 0.87 (0.58, 1.30) 0.65 (0.28, 1.55)	1 0.99 (0.69, 1.40) 0.79 (0.50, 1.23) 1.05 (0.49, 2.27)
AH4 score (literacy/numeracy measure)	0.98 (0.97, 1.00)	0.97 (0.96, 0.99)**	0.99 (0.97, 1.00)
Rosenberg self-esteem score	0.98 (0.94, 1.02)	0.98 (0.94, 1.02)	1.01 (0.96, 1.06)
Number community clubs involved in the following:			
None One Two Three Four or more	1 1.22 (0.90, 1.65) 0.98 (0.65, 1.49) 1.09 (0.59, 2.00) 1.22 (0.57, 2.59)	1 1.01 (0.77, 1.32) 0.81 (0.58, 1.12) 1.03 (0.69, 1.55) 0.97 (0.58, 1.65)	1 0.96 (0.69, 1.32) 1.50 (1.02, 2.21)* 0.85 (0.51, 1.43) 1.49 (0.86, 2.58)
Not limited by LTC Limiting longstanding illness	1 1.15 (0.96, 1.34)	1 1.28 (0.98, 1.68)	1.01 (0.75, 1.37)
Maximum educational achievement by age 35			
None Standard grade Apprenticeship Higher HND Degree	1 1.82 (1.16, 2.84)** 1.46 (1.02, 2.07)* 1.34 (0.80, 2.26) 1.99 (0.75, 5.32) 1.49 (0.91, 2.45)	1 1.52 (1.04, 2.22)** 0.96 (0.68, 1.34) 1.24 (0.76, 2.00) 2.69 (1.39, 5.23)** 1.52 (0.98, 2.37)	1 1.32 (0.85, 2.07) 1.34 (0.87, 2.07) 1.06 (0.59, 1.89) 1.69 (0.85, 3.37) 0.90 (0.53, 1.52)
Health locus of control statements			
I have the power to make myself well Agree strongly Agree quite a bit Agree a little Disagree a little Disagree quite a bit Disagree a lot	1 1.25 (0.78, 2.00) 0.84 (0.51, 1.37) 0.67 (0.37, 1.19) 0.87 (0.46, 1.67) 0.58 (0.30, 1.10)	1 1.06 (0.72, 1.57) 0.67 (0.45, 1.01) 0.53 (0.32, 0.89)** 0.67 (0.39, 1.15) 0.52 (0.29, 0.93)**	1 0.96 (0.56, 1.65) 0.76 (0.44, 1.32) 0.96 (0.50, 1.85) 0.82 (0.40, 1.70) 0.76 (0.37, 1.57)
I have no control over being ill			
Agree strongly Agree quite a bit Agree a little Disagree a little Disagree quite a bit Disagree a lot	1 0.46 (0.25, 0.86)** 0.76 (0.42, 1.38) 0.98 (0.53, 1.82) 0.60 (0.33, 1.08) 0.95 (0.55, 1.64)	1 1.02 (0.59, 1.77) 1.26 (0.74, 2.14) 1.63 (0.94, 2.84) 1.25 (0.73, 2.12) 1.32 (0.80, 2.16)	1 0.87 (0.45, 1.67) 0.77 (0.40, 1.50) 1.07 (0.54, 2.11) 0.48 (0.25, 0.94)* 0.75 (0.40, 1.43)
Regular doctor visits reduce health			
Agree strongly Agree quite a bit Agree a little Disagree a little Disagree quite a bit Disagree a lot	1 2.58(1.41, 4.70)** 1.37 (0.81, 2.32) 1.03 (0.55, 1.92) 0.92 (0.51, 1.68) 1.09 (0.62, 1.92)	1 1.71 (1.03, 2.83) 1.08 (0.69, 1.69) 0.81 (0.48, 1.39) 0.95 (0.58, 1.56) 0.88 (0.55, 1.40)	1 1.70 (0.82, 3.53) 1.56 (0.78, 3.13) 0.93 (0.44, 1.97) 1.17 (0.59, 2.35) 1.31 (0.65, 2.61)
Accidental happening influence health			
Agree strongly Agree quite a bit Agree a little Disagree a little Disagree quite a bit Disagree a lot	1 1.44 (0.70, 2.96) 1.52 (0.79, 2.91) 1.45 (0.74, 2.85) 2.22 (1.12, 4.38)* 1.20 (0.63, 2.29)	1 1.52 (0.80, 2.89) 1.11 (0.62, 2.00) 1.41 (0.76, 2.61) 1.29 (0.70, 2.38) 1.02 (0.57, 1.82)	1 1.36 (0.60, 3.07) 0.91 (0.43, 1.92) 1.17 (0.53, 2.54) 1.01 (0.48, 2.11) 1.66 (0.79, 3.47)
Only doctors can maintain health			
Agree strongly Agree quite a bit Agree a little Disagree a little Disagree quite a bit Disagree a lot	1 1.11 (0.57, 2.21) 0.55 (0.28, 1.10) 0.51 (0.25, 1.04) 0.66 (0.34, 1.34) 0.78 (0.41, 1.51)	1 1.29 (0.70, 2.39) 0.58 (0.31, 1.08) 0.71 (0.38, 1.33) 0.87 (0.47, 1.61) 0.99 (0.55, 1.77)	1 1.26 (0.51, 3.09) 0.94 (0.40, 2.24) 1.89 (0.82, 4.37) 1.90 (0.83, 4.32) 1.67 (0.73, 3.79)
I am responsible for my health			
Agree strongly Agree quite a bit Agree a little Disagree a little Disagree quite a bit Disagree a lot	1 0.99 (0.68, 1.43) 1.42 (0.90, 2.22) 1.45 (0.82, 2.59) 1.45 (0.64, 3.30) 1.08 (0.48, 2.42)	1 1.09 (0.79, 1.52) 1.65 (1.11, 2.45)** 1.49 (0.90, 2.47) 1.21 (0.61, 2.40) 0.63 (0.30, 1.32)	1 1.10 (0.75, 1.62) 1.22 (0.75, 1.97) 0.47 (0.22, 1.02) 1.39 (0.62, 3.13) 0.77 (0.31, 1.86)
Others are responsible for my health			
Agree strongly Agree quite a bit Agree a little Disagree a little Disagree quite a bit Disagree a lot	1 0.84 (0.44, 1.59) 0.78 (0.42, 1.44) 1.14 (0.59, 2.20) 0.69 (0.38, 1.28) 0.96 (1.04, 1.65)	1 1.21 (0.68, 2.17) 0.84 (0.48, 1.48) 1.68 (0.94, 3.01) 0.78 (0.45, 1.34) 1.17 (0.70, 1.96)	1 1.71 (0.73, 3.99) 1.70 (0.78, 3.70) 1.97 (0.88, 4.41) 1.55 (0.72, 3.36) 1.68 (0.81, 3.47)
Its my fault when things go wrong with my health			
Agree strongly Agree quite a bit Agree a little Disagree a little Disagree quite a bit Disagree a lot	1 1.23 (0.71, 2.13) 0.99 (0.58, 1.71) 1.02 (0.59, 1.75) 1.03 (0.59, 1.79) 0.95 (0.55, 1.63)	1 1.23 (0.75, 2.02) 1.35 (0.84, 2.18) 1.33 (0.81, 2.19) 1.45 (0.87, 2.40) 1.14 (0.69, 1.88)	1 0.74 (0.38, 1.41) 0.87 (0.47, 1.63) 0.75 (0.38, 1.46) 1.21 (0.64, 2.29) 0.84 (0.43, 1.66)
When I am ill, I let nature run its course			
Agree strongly Agree quite a bit Agree a little Disagree a little Disagree quite a bit Disagree a lot	1 0.91 (0.51, 1.63) 1.05 (0.63, 1.77) 0.94 (0.52, 1.68) 1.22 (0.70, 2.11) 1.67 (0.97, 1.85)	1 1.09 (0.68, 1.76) 1.04 (0.67, 1.62) 1.12 (0.67, 1.85) 1.05 (0.65, 1.69) 1.07 (0.67, 1.70)	1 0.66 (0.36, 1.23) 1.05 (0.59, 1.87) 1.13 (0.60, 2.14) 0.92 (0.50, 1.70) 1.06 (0.59, 1.89)
When I am healthy its because I am lucky			
Agree strongly Agree quite a bit Agree a little Disagree a little Disagree quite a bit Disagree a lot	1 0.53 (0.27, 1.03) 0.80 (0.42, 1.54) 0.64 (0.34, 1.26) 0.80 (0.42, 1.57) 0.92 (0.46, 1.83)	1 0.78 (0.46, 1.34) 0.94 (0.55, 1.62) 0.85 (0.49, 1.49) 0.95 (0.53, 1.71) 1.06 (0.59, 1.91)	1 0.71 (0.36, 1.41) 0.69 (0.35, 1.35) 0.53 (0.26, 1.07) 0.61 (0.31, 1.20) 0.66 (0.32, 1.34)
Wellbeing depends on taking care of yourself			
Agree strongly Agree quite a bit Agree a little Disagree a little Disagree quite a bit Disagree a lot	1 1.10 (0.66, 1.84) 1.20 (0.72, 1.99) 1.36 (0.75, 2.46) 1.23 (0.68, 2.22) 0.92 (0.52, 1.63)	1 1.00 (0.64, 1.57) 1.10 (0.71, 1.71) 1.13 (0.68, 1.85) 1.14 (0.70, 1.85) 0.75 (0.46, 1.24)	1 1.21 (0.65, 2.26) 0.98 (0.51, 1.88) 1.21 (0.60, 2.45) 0.97 (0.51, 1.83) 1.05 (0.54, 2.05)
Illness means you have not cared for yourself			
Agree strongly Agree quite a bit Agree a little Disagree a little Disagree quite a bit Disagree a lot	1 1.16 (0.81, 1.67) 1.16 (0.71, 1.90) 1.04 (0.39, 2.73) 0.34 (0.12, 0.96) 0.95 (0.34, 2.62)	1 1.05 (0.77, 1.44) 1.22 (0.80, 1.85) 1.72 (0.85, 3.50) 0.68 (0.26, 1.77) 1.98 (0.83, 4.73)	1 1.05 (0.73, 1.50) 1.42 (0.87, 2.31) 0.98 (0.39, 2.46) 0.64 (0.20, 2.01) 1.32 (0.60, 2.90)
Care from others helps me to get well			
Agree strongly Agree quite a bit Agree a little Disagree a little Disagree quite a bit Disagree a lot	1 0.77 (0.48, 1.25) 0.74 (0.45, 1.23) 0.52 (0.30, 0.89) 0.82 (0.47, 1.42) 0.76 (0.42, 1.36)	1 0.48 (0.32, 0.73)** 0.61 (0.40, 0.92)** 0.41 (0.25, 0.65)** 0.53 (0.33, 0.86)** 0.76 (0.46, 1.26)	1 0.69 (0.40, 1.20) 1.08 (0.62, 1.88) 0.83 (0.46, 1.51) 0.79 (0.42, 1.46) 0.98 (0.52, 1.84)
Illness is luck			
Agree strongly Agree quite a bit Agree a little Disagree a little Disagree quite a bit Disagree a lot	1 0.64 (0.41, 1.01) 0.66 (0.42, 1.04) 1.28 (0.73, 2.22) 0.62 (0.32, 1.20) 0.59 (0.29, 1.22)	1 0.87 (0.59, 1.29) 1.11 (0.74, 1.65) 1.45 (0.89, 2.36) 0.97 (0.56, 1.70) 0.92 (0.51, 1.66)	1 1.08 (0.65, 1.80) 0.80 (0.48, 1.36) 0.71 (0.39, 1.32) 0.97 (0.49, 1.90) 1.08 (0.51, 2.29)
Looking after myself keeps me healthy			
Agree strongly Agree quite a bit Agree a little Disagree a little Disagree quite a bit Disagree a lot	1 0.98 (0.58, 1.68) 1.38 (0.81, 2.35) 1.01 (058, 1.79) 1.64 (0.93, 2.92) 1.86 (1.06, 3.27)*	1 1.04 (0.66, 1.65) 1.29 (0.82, 2.01) 0.82 (0.50, 1.36) 1.17 (0.73, 1.88) 1.61 (0.97, 2.66)	1 0.93 (0.54, 1.60) 1.14 (0.66, 1.98) 0.86 (0.47, 1.58) 1.10 (0.63, 1.93) 0.88 (0.46, 1.66)
Doctor’s orders keep me healthy			
Agree strongly Agree quite a bit Agree a little Disagree a little Disagree quite a bit Disagree a lot	1 0.77 (0.37, 1.61) 0.76 (0.38, 1.52) 0.50 (0.25, 1.03) 0.58 (0.29, 1.18) 0.65 (0.32, 1.31)	1 0.76 (0.40, 1.44) 0.77 (0.43, 1.40) 0.62 (0.33, 1.14) 0.67 (0.36, 1.25) 0.95 (0.52, 1.73)	1 0.73 (0.32, 1.64) 0.85 (0.39, 1.82) 0.98 (0.45, 2.14) 0.89 (0.41, 1.92) 0.89 (0.41, 1.94)
I can usually stay healthy by taking good care of myself			
Agree strongly Agree quite a bit Agree a little Disagree a little Disagree quite a bit Disagree strongly	1 1.17 (0.78, 1.74) 1.47 (0.93, 2.33) 1.77 (0.95, 3.30) 2.62 (1.19, 5.98)* 1.89 (0.60, 1.13)	1 1.2 (0.84, 1.71) 1.18 (0.77, 1.80) 1.25 (0.72, 2.18) 1.50 (0.77, 2.91) 1.02 (0.39, 2.64)	1 1.08 (0.72, 1.63) 0.87 (0.52, 1.44) 0.94 (0.48, 1.86) 0.83 (0.36, 1.94) 0.51 (0.15, 1.72)
Following the doctors order on the letter is the way to stay healthy			
Agree strongly Agree quite a bit Agree a little Disagree a little Disagree quite a bit Disagree strongly	1 0.76 (0.52, 1.14) 0.76 (0.50, 1.17) 0.41 (0.22, 0.76)** 1.01 (0.54, 1.89) 0.29 (0.12, 0.63)**	1 0.81 (0.57, 1.14) 1.02 (0.70, 1.49) 0.58 (0.34, 0.98)* 1.16 (0.68, 1.97) 0.52 (0.27, 0.98)*	1 1.00 (0.65, 1.53) 0.80 (0.47, 1.35) 1.04 (0.58, 1.86) 0.78 (0.43, 1.43) 0.53 (0.24, 1.15)
Mastery statements			
I have little control over what happens to me Strongly agree Agree Disagree Strongly disagree	1 0.59 (0.25, 1.37) 0.57 (0.25, 1.31) 0.50 (0.20, 1.25)	1 0.52 (0.27, 0.99)* 0.48 (0.25, 0.91) * 0.56 (0.28, 1.14)	1 2.02 (0.77, 5.33) 2.07 (0.79, 5.40) 1.44 (0.53, 3.89)
There is no way I can solve some of the problems I have			
Strongly agree Agree Disagree Strongly disagree	1 1.07 (0.45, 2.54) 0.98 (0.40, 2.41) 1.43 (0.55, 3.71)	1 0.88 (0.45, 1.72) 0.81 (0.40, 1.64) 0.80 (0.37, 1.75)	1 0.86 (0.34, 2.13) 0.64 (0.25, 1.64) 0.54 (0.19, 1.53)
There is little I can do to change many of the important things in my life			
Strongly agree Agree Disagree Strongly disagree	1 0.49 (0.18, 1.31) 0.50 (0.18, 1.38) 0.57 (0.19, 1.71)	1 0.62 (0.30, 1.29) 0.58 (0.27, 1.24) 0.52 (0.22, 1.21)	1 0.51 (0.20, 1.30) 0.56 (0.21, 1.51) 0.58 (0.20, 1.71)
Sometimes I feel helpless dealing with the problems in life			
Strongly agree Agree Disagree Strongly disagree	1 0.71 (0.28, 1.78) 0.79 (0.31, 1.97) 0.53 (0.20, 1.39)	1 1.15 (0.55, 2.41) 1.25 (0.58, 2.71) 1.09 (0.47, 2.49)	1 2.37 (0.83, 6.74) 3.20 (1.07, 9.51)* 3.32 (1.07, 10.26)*
Sometimes I feel I am pushed around in life			
Strongly agree Agree Disagree Strongly disagree	1 0.59 (0.24, 1.48) 0.93 (0.38, 2.29) 0.54 (0.20, 1.41)	1 0.94 (0.43, 2.05) 1.48 (0.67, 3.26) 1.26 (0.54, 2.96)	1 0.71 (0.3, 1.69) 0.67 (0.28, 1.63) 0.76 (0.29, 1.97)
What happens in the future depends mostly on me			
Strongly agree Agree Disagree Strongly disagree	1 1.20 (0.75, 1.91) 1.23 (0.71, 2.13) 0.78 (0.35, 1.71)	1 1.02 (0.68, 1.52) 1.17 (0.72, 1.89) 0.54 (0.27, 1.10)	1 0.65 (0.42, 1.02) 0.44 (0.25, 0.78)** 0.64 (0.26, 1.57)
I can do just about anything I set my mind to			
Strongly agree Agree Disagree Strongly disagree	1 0.75 (0.46, 1.21) 0.66 (0.37, 1.17) 1.06 (0.45, 2.52)	1 0.98 (0.64,1.51) 0.83 (0.50, 1.39) 1.34 (0.62, 2.91)	1 0.89 (0.55, 1.42) 0.97 (0.54, 1.72) 1.42 (0.56, 3.58)

^*^p < 0.05. **p < 0.01

^a^Adjusted for age, sex, alcohol, exercise, fruit and vegetable intake, BMI, smoking, marital status, blood pressure and number of long-term conditions. In addition, the presented results for each capacity variable are also adjusted for the other capacity variables in the expressing group (e.g. the row for self-assessed health results has been adjusted for self-assessed health compared to others your age, disability, depression, anxiety, literacy, self-esteem, involvement in community clubs, education, health locus of control and mastery statements)

^b^Numbers in this category too small to allow estimation of HR

There was an association between registered disability and both outcome measures. Poor self-rated health for your age was strongly associated with mortality (but not hospitalisation) while not rating your health as good over the last year was strongly associated with hospitalisation (but not mortality). For the health locus of control and mastery statements, there were several isolated associations for one reply within a statement, which would have been expected by chance.

However, disagreeing with the statement “care from others helps me get well”, or “I have little control over what happens to me”, was associated with decreased mortality. Meanwhile, disagreeing with “sometimes I feel helpless dealing with the problems in life” was associated with hospitalisation. This may suggest a protective mortality association for those who are more independent or feel a higher sense of control over their multimorbidity, who may, in turn, also be more likely to attend hospital.

#### Community capacity (Table 4)

**Table 4:** Relationship between community capacity construct variables, mortality and hospitalisation

**Variables**	**Hazard ratio (95% confidence interval) for mortality**		**Odds ratio for ** **self-reported hospital admission in the last year** **(95% confidence interval)**
	Baseline model^a^ (using baseline covariates)	Time-varying model^a^ (adjusting for time-varying covariates)	
Exchange small favours with those who live nearby			
Yes	1	1	1
No	0.96 (0.88, 1.23)	1.02 (0.86, 1.19)	0.96 (0.79, 1.18)
How do you feel about the area you live in (faces scale)			
1 — most positive 2 3 4 5 6 7 — most negative	1 0.97 (0.82, 1.16) 0.87 (0.70, 1.07) 0.88 (0.65, 1.20) 0.73 (0.46, 1.15) 1.07 (0.56, 2.03) 1.31 (0.85, 2.01)	1 0.83 (0.71, 0.98)* 0.88 (0.73, 1.06) 0.98 (0.74, 1.29) 0.80 (0.51, 1.27) 1.08 (0.66, 1.75) 1.59 (1.02, 2.41)*	1 1.15 (0.93, 1.43) 1.23 (0.95, 1.58) 1.01 (0.70, 1.46) 1.02 (0.58, 1.79) 2.00 (1.07, 3.73)** 1.28 (0.69, 2.36)
How do you feel about walking around the area after dark? Would you say that you			
Never Try to avoid Feel uncomfortable Have no worries	1 0.94 (0.73, 1.20) 0.89 (0.68, 1.15) 0.98 (080, 1.20)	1 0.74 (0.60, 0.92)** 0.70 (0.55, 0.89)** 0.69 (0.57, 0.83)**	1 0.85 (0.63, 1.13) 0.64 (0.47, 0.89)** 0.79 (0.61, 1.02)
Around the area you live, would you say vandalism is a problem?			
Serious problem Minor problem No problem	1 0.86 (0.68, 1.08) 0.95 (0.74, 1.23)	1 1.03 (0.82, 1.30) 1.00 (0.78, 1.29)	1 0.98 (0.73, 1.31) 1.01 (0.74, 1.39)
Around the area you live, would you say litter is a problem?			
Serious problem Minor problem No problem	1 1.04 (0.83, 1.29) 0.94 (0.75, 1.19)	1 1.02 (0.83, 1.25) 1.19 (0.89, 1.36)	1 1.01 (0.78, 1.30) 1.15 (0.87, 1.53)
Around the area you live, would you say assaults are a problem?			
Serious problem Minor problem No problem	1 1.11 (0.85, 1.44) 0.95 (0.73, 1.24)	1 0.94 (0.72, 1.24) 0.89 (0.68, 1.17)	1 0.64 (0.45, 0.89)** 0.64 (0.45, 0.91)*
Around the area you live, would you say burglaries are a problem?			
Serious problem Minor problem No problem	1 1.07 (0.89, 1.29) 1.20 (0.97, 1.49)	1 0.92 (0.74, 1.15) 1.02 (0.82, 1.28)	1 1.11 (0.87, 1.41) 1.19 (0.93, 1.54)
Around the area you live, would you say young people causing disturbances are a problem?			
Serious problem Minor problem No problem	1 1.28 (0.94, 1.74) 1.10 (0.82, 1.49)	1 1.04 (0.80, 1.37) 1.03 (0.78, 1.35)	1 1.31 (0.93, 1.86) 1.13 (0.79, 1.60)

^*^p < 0.05. **p < 0.01

^a^Adjusted for age, sex, alcohol, exercise, fruit and vegetable intake, BMI, smoking, marital status, blood pressure and number of long-term conditions. In addition, the presented results for each capacity variable are also adjusted for the other capacity variables in the community group (e.g. the row for exchange small favour results has been adjusted for how you feel about the area, how you feel about walking in the dark and whether you feel vandalism, litter, assaults, burglaries or young people causing disturbances is a problem in your area)

People who never walked around their community after the dark had the highest mortality hazard ratios. Concern about problems (such as vandalism or litter) in the community was not associated with mortality, although serious concern about assaults in the community was associated with increased hospitalisation.

Finally, capacity factors change over time and accommodating that change within the analysis changes the ratios compared to simply using a baseline measure. For example, the associations between mortality and walking in the dark, self-assessed health for “someone your own age”, and loneliness were only clear when time-varying covariates were used.

### The influence of socioeconomic deprivation

An initial exploratory test of potential moderation of SED was undertaken by splitting the cohort into high (more deprived) and low (more affluent) subsets. SED was taken out of the model, and then the community capacity model was run on the two subsets (Table 5).

**Table 5:** Mortality and hospitalisation for the community capacity construct variables for the whole data set and the high and low DEPCAT subsets

**Variables**	**Hazard ratio for mortality (95% confidence interval) adjusted for confounders**^**a**^			**Odds ratio for self-reported hospital admission in the last year (95% confidence interval) adjusted for confounders**^**a**^		
	Whole dataset	Affluent subset (***n*** = 734)	Deprived subset (***n*** = 3733)	Whole dataset	Affluent subset (***n*** = 734)	Deprived subset (***n*** = 3733)
**Exchange small favours with those who live nearby**						
**Yes** **No**	1 1.03 (0.88,1.21)	1 0.92 (0.53, 1.60)	1 0.98 (0.85, 1.14)	1 0.96 (0.79, 1.16)	1 1.68 (0.92, 3.29)	1 0.87 (0.71, 1.07)
**How do you feel about the area you live in (faces scale)**						
**1 — most positive** **2** **3** **4** **5** **6** **7 — most negative**	1 0.86 (0.73,1.02) 0.91 (0.75, 1.1) 1.03 (0.78,1.35) 0.76 (0.48,1.21) 1.18 (0.72,1.92) 1.58 (1.04,2.39)*	1 0.87 (0.58, 1.29) 0.52 (0.27, 0.97)* 1.12 (0.45, 2.82) 0.57 (0.07, 4.71) 4.61 (0.5, 42.78) -	1 0.92 (0.78, 1.08) 0.97 (0.81, 1.16) 1.03 (0.80, 1.33) 0.70 (0.45, 1.07) 1.22 (0.76, 1.96) 1.54 (1.06, 2.25)*	1 1.17 (0.95, 1.43) 1.25 (0.99, 1.58) 1.01 (0.70, 1.44) 1.00 (0.58, 1.65) 1.96 (1.09, 3.39)* 1.18 (0.64, 2.08)	1 1.10 (0.69, 1.74) 1.42 (0.80, 2.47) 0.61 (0.13, 2.00) - - -	1 1.23 (0.98, 1.56) 1.26 (0.97, 1.64) 1.09 (0.74, 1.59) 1.11 (0.64, 1.86) 2.19 (1.20, 3.85)** 1.21 (0.65, 2.16)
**How do you feel about walking around the area after dark? Would you say that you**						
**Never** **Try to avoid** **Feel uncomfortable** **Have no worries**	1 0.70 (0.57, 0.87)** 0.66 (0.52, 0.84)** 0.63 (0.53, 0.76)**	1 0.89 (0.45, 1.75) 0.86 (0.39, 1.89) 0.77 (0.41, 1.43)	1 0.65 (0.53, 0.80)** 0.62 (0.49, 0.79)** 0.66 (0.55, 0.78)**	1 0.80 (0.60, 1.05) 0.61 (0.45, 0.83)** 0.71 (0.55, 0.91)**	1 0.73 (0.35, 1.58) 0.42 (0.17, 1.01) 0.76 (0.39, 1.53)	1 0.77 (0.57, 1.04) 0.63 (0.45, 0.87)** 0.68 (0.52, 0.89)**
**Around the area you live, would you say vandalism is a problem?**						
**Serious problem** **Minor problem** **No problem**	1 1.03 (0.82, 1.30) 1.01 (0.78, 1.30)	1 0.77 (0.31, 1.92) 0.66 (0.25, 1.73)	1 1.14 (0.93, 1.40) 1.18 (0.94, 1.50)	1 0.96 (0.73, 1.27) 0.99 (0.73, 1.35)	1 0.42 (0.15, 1.27) 0.44 (0.15, 1.37)	1 1.01 (0.76, 1.36) 1.07 (0.78, 1.49)
**Around the area you live, would you say litter is a problem?**						
**Serious problem** **Minor problem** **No problem**	1 1.01 (0.83, 1.24) 1.12 (0.90, 1.39)	1 0.64 (0.35, 1.19) 0.87 (0.47, 1.61)	1 0.96 (0.80, 1.16) 1.06 (0.87, 1.30)	1 1.01 (0.79, 1.30) 1.17 (0.90, 1.53)	1 1.99 (0.84, 5.37) 1.75 (0.71, 4.84)	1 0.93 (0.71, 1.21) 1.13 (0.85, 1.50)
**Around the area you live, would you say assaults are a problem?**						
**Serious problem** **Minor problem** **No problem**	1 0.98 (0.74, 1.29) 0.92 (0.70, 1.21)	1 0.85 (0.10, 7.32) 1.03 (0.13, 8.51)	1 0.91 (0.71, 1.15) 0.84 (0.66, 1.06)	1 0.63 (0.45, 0.87)** 0.63 (0.45, 0.87)**	1 3.25 (0.45, 73.11) 3.21 (0.46, 70.81)	1 0.60 (0.43, 0.85)** 0.59 (0.42, 0.84)**
**Around the area you live, would you say burglaries are a problem?**						
**Serious problem** **Minor problem** **No problem**	1 0.93 (0.75, 1.16) 1.05 (0.84, 1.31)	1 1.82 (0.74, 4.49) 2.10 (0.83, 5.31)	1 0.86 (0.71, 1.04) 0.92 (0.75, 1.12)	1 1.14 (0.90, 1.45) 1.26 (0.98, 1.62)	1 0.95 (0.52, 1.80) 1.16 (0.62, 2.24)	1 1.17 (0.90, 1.52) 1.28 (0.98, 1.69)
**Around the area you live, would you say young people causing disturbances are a problem?**						
**Serious problem** **Minor problem** **No problem**	1 1.01 (0.77, 1.32) 0.98 (0.75, 1.29)	1 - -	1 1.10 (0.86, 1.41) 1.00 (0.79, 1.28)	1 1.20 (0.86, 1.69) 1.02 (0.73, 1.44)	1 0.82 (0.23, 3.97) 0.9 0(0.26, 4.33)	1 1.27 (0.90, 1.82) 1.03 (0.73, 1.47)

^*^p < 0.05. **p < 0.01

^a^Adjusted for age, sex, alcohol, exercise, fruit and vegetable intake, BMI, smoking, marital status, blood pressure and number of long-term conditions

The cohort has a negative socio-economic deprivation skew because it was drawn from the West of Scotland which has a more deprived profile than the rest of Scotland. The resulting small numbers for some variables (feelings about community, concerns about young people causing disturbances) in the affluent subset meant OR and HR were not able to be estimated. The subset analysis showed the significant positive association with concerns about walking in the dark and assaults disappeared in the more affluent subset while becoming stronger in the deprived subset for both mortality and hospital admissions.

## Discussion

This is the first time, to our knowledge, that any attempt has been made to try and quantify capacity factors and measure their association with mortality and hospital admissions in the context of multimorbidity. We have shown that BOTT provides a structure to explore capacity variables in population cohorts and that capacity measures vary over time (not adjusting for this in the analysis could potentially over-estimate or miss associations). Positive associations were seen with both types of individual (mobilising and expressing) and community factors; this is in keeping with the underlying tenets of BOTT. Capacity is complex and multifactorial. Initial exploratory analysis suggests SED could be a potential moderator of these capacity factors, which requires further investigation.

This was an exploratory analysis, seeking to understand if it was possible to start to measure factors that might impact capacity. Therefore, further work to clarify and explore these potential relationships in more detail, and in other cohort populations, is warranted.

### Comparison with other literature

While registered disability has been associated with mortality in the context of older populations with multimorbidity [44], our findings demonstrate this association is present even when adjusting for age and for the first time have shown it is also associated with hospitalisation. The persistent strong association between feeling unsafe walking in the dark in a community and mortality is an important novel finding, especially when other feelings about the community did not appear to have an association.

This work highlighted an association between the extent to which people feel they have control over their health and both mortality and hospitalisation, although the exact relationship is not clear. Previous literature has shown that having a higher external locus of control is associated with both the prevalence of multimorbidity [58–60] and the development of it in over 50 s [61]. It is not clear whether this relationship is a cause or consequence [60]; this work adds to the body of evidence that it should be explored further.

Variables such as anxiety and depression, or number of social contacts, which we might have expected to influence outcomes, did not show any association. Sharing feelings appeared to be protective for mortality, but the similar capacity factor of having someone to confide in showed no association. Other work has shown that emotional social support is protective for mortality, reduces deterioration in psychological comorbidities and reduces non-adherence with treatment in people with multimorbidity [62–64]. These studies used validated questionnaires, rather than one-off questions, and it may be that further work looking at more precise measurement will clarify this relationship more.

The number of social contacts appeared to have little impact, but this could be because detailed information was only measured in waves 1–3, and there was simply insufficient data available to explore the relationship adequately.

Multimorbidity is closely associated with frailty (particularly important in younger adults), and there is a bidirectional relationship between the two [65–67]. However, while related, multimorbidity and frailty are recognised and should be considered as two different concepts [67]. Therefore, the analysis focused on multimorbidity. It is expected that there will be complex interactions between capacity factors, multimorbidity and frailty, and this could be explored in future work.

Capacity is an understudied construct, and at present, there are no validated measures [20]. One potential suggested measure is the Patient Activation Measure which examines how “activated” patients are in terms of engagement with their care [68]. However, while similar, it does not fully capture capacity which influences patient experience regardless of health service engagement and does not take account of community factors [20]. Another suggestion is a measure of “flourishing” [69], whose constructs align with some capacity constructs [20]. However, it has methodological weaknesses and was based on a healthy population, meaning its utility in those with multimorbidity remains uncertain [20, 69]. Neither measure therefore is fit for purpose to measure capacity in people with multimorbidity.

We were not able to look at the construct of treatment burden directly as the cohort did not contain variables that distinguished between illness and treatment burden. However, there are validated treatment burden measures; hopefully, if used in future cohorts, the relationship between treatment burden and capacity factors (to one another and to wider outcomes) could be explored. Future work could build on the current study to explore better the role of SED and to build a capacity measure that could inform the design and evaluation of future multimorbidity interventions and services.

### Strengths and limitations

A key strength of this exploratory analysis is that it is the first time, to our knowledge, anyone has attempted to measure multiple capacity factors (individual and community) and potential associations with mortality and hospitalisation in the context of multimorbidity. Due to the complex, multifactorial interactions of these factors, this is a key strength. A further strength is that the analysis, and variable choices, is underpinned by a recognised theory, BOTT. Given the impact of SED on BOTT constructs, another strength is that, unlike most research cohorts, the WoS20-07 was recruited specifically to have as representative a population as possible, resulting in skewness in the SED distribution of a more deprived rather than affluent population. In addition, the cohort had four waves of data collection, as well as baseline factors, allowing the analysis to adjust for change in factors over time.

The key limitation of this analysis was the structure of the cohort itself. Like most cohorts, there was attrition over each wave as the study progressed. However, there were also different questions asked across different waves, making it difficult to measure the same variable over time, requiring proxies to be used in certain waves. In addition, in the earlier waves, some questions were only asked of the older cohorts.

Given the large number of variables, one would expect some positive findings by chance, and this should be considered when interpreting the results. As stated, this is an exploratory analysis, and findings need to be replicated in other cohorts to clarify potential associations. In addition, cohorts that may have more precise measures of capacity variables such as emotional and social support will allow their impact to be better examined in people with multimorbidity. It should also be noted that the community variables that were available were subjective (how people felt about their neighbourhood). Future work should consider cohorts including neighbourhood measures (e.g. local amenities, green space).

As mentioned, the cohort included people who had multimorbidity at any time within the follow-up. While the sensitivity analysis did not show any significant impact in effect size when accounting for the presence of multimorbidity as a time-varying variable, it should be noted that those who developed multimorbidity later had fewer years of follow-up, which is a limitation of the study. In addition, although age was a covariate in the model, future research on larger data sets could use age-stratified or interaction analyses to study whether the impact of capacity differs by age group.

Finally, the considerable number of social contact questions asked in the earlier waves were replaced in the last two waves with one binary question. This meant that the influence of the number, or type of social networks (shown to be important in other literature [38, 39]), could not be explored in any depth. Further, the cohort is 98.1% white, which was reflective of the West of Scotland at the start of data collection. A final limitation is the timing of the data collection — it measured data from the 1980 s to 2007, and society has changed in important ways since then. The ethnicity of Scotland has changed and could be expected to be a potential confounder. In addition, our understanding of chronic disease, mental health and concepts such as loneliness has also changed and might influence results. This cohort will also not cover the impact of recent technology such as smart phones and health apps.

## Conclusions

This paper describes a novel analysis that shows BOTT provides a structure allowing individual and community capacity constructs to be measured and highlights the importance of adjusting for how they vary over time. Factors ranging from literacy and tenancy to feeling unsafe walking in the dark in your community and level of control people feel they have over their lives have been shown to be associated with both hospitalisations and mortality.

Future work could build on these findings to explore the role of SED on capacity factors and whether these associations, or others, are found in other cohort populations. This could inform future development of a measure of capacity. Our work contributes to the growing body of evidence that capacity issues, such as housing, may be important when designing future multimorbidity interventions and services.

## Supplementary Information

Supplementary material 1. Figure S1: Mobilising Capacity DAG. Figure S2: Expressing Capacity DAG. Figure S3: Community Capacity DAG. Table S1: Conditions used to identify Multimorbidity. Table S2: Mobilising Capacity variables. Table S3: Expressing Capacity Variable. Table S4: community Capacity Variables. Table S5: Table demonstrating Hazard Ratio for Mobilising Capacity Variables when not adjustingor adjustingfor presence of absence of multimorbidity. Table S6: Table demonstrating Odds Ratio for self-reported hospital admission for Mobilising Capacity Variables when not adjustingor adjustingfor presence of absence of multimorbidity. Table S7: Table demonstrating Hazard Ratio for Expressing Capacity Variables when not adjustingor adjustingfor presence of absence of multimorbidity. Table S8: Table demonstrating Odds Ratio for self-reported hospital admission for Expressing Capacity Variables when not adjustingor adjustingfor presence of absence of multimorbidity. Table S9: demonstrating Hazard Ratio for Community Capacity Variables when not adjustingor adjustingfor presence of absence of multimorbidity. Table S10 demonstrating Odds Ratio for self-reported hospital admission for Mobilising Capacity Variables when not adjustingor adjustingfor presence of absence of multimorbidity.

## Data Availability

The data are employed here with the permission of the Twenty-07 Steering Group. We welcome applications from bona fide researchers to share the data in collaboration with the study team. Email sphsu-datasharing@glasgow.ac.uk, quoting The West of Scotland Twenty-07 study, for further information.

## Abbreviations

BOTT: Burden of Treatment Theory
DAG: Directed acyclic graphs
SED: Socioeconomic deprivation
MLTC: Multiple long-term conditions
DEPCAT: Standardised Deprivation Categories used with the Carstairs score
MICE: Multivariable imputation by chained equations
MAR: Data missing at random

## References

[1] Barnett K, Mercer SW, Norbury M, Watt G, Wyke S, Guthrie B. Epidemiology of multimorbidity and implications for health care, research, and medical education: a cross-sectional study. Lancet. 2012;380(9836):37–43. 22579043 10.1016/S0140-6736(12)60240-2

[2] Skou ST, Mair FS, Fortin M, Guthrie B, Nunes BP, Miranda JJ, et al. Multimorbidity. Nat Rev Dis Primers. 2022;8(1): 48. 35835758 10.1038/s41572-022-00376-4 PMC7613517

[3] Ryan BL, Bray Jenkyn K, Shariff SZ, Allen B, Glazier RH, Zwarenstein M, et al. Beyond the grey tsunami: a cross-sectional population-based study of multimorbidity in Ontario. Can J Public Health. 2018;109(5):845–54. 30022403 10.17269/s41997-018-0103-0 PMC6964436

[4] Asogwa OA, Boateng D, Marzà-Florensa A, Peters S, Levitt N, Olmen Jv, et al. Multimorbidity of non-communicable diseases in low-income and middle-income countries: a systematic review and meta-analysis. BMJ Open. 2022;12(1): e049133. 35063955 10.1136/bmjopen-2021-049133 PMC8785179

[5] Price AJ, Jobe M, Sekitoleko I, Crampin AC, Prentice AM, Seeley J, et al. Epidemiology of multimorbidity in low-income countries of sub-Saharan Africa: findings from four population cohorts. PLoS Glob Public Health. 2023;3(12): e0002677. 38055698 10.1371/journal.pgph.0002677 PMC10699623

[6] Krauth SJ, Steell L, Ahmed S, McIntosh E, Dibben GO, Hanlon P, et al. Association of latent class analysis-derived multimorbidity clusters with adverse health outcomes in patients with multiple long-term conditions: comparative results across three UK cohorts. EClinicalMedicine. 2024;74: 102703. 39045545 10.1016/j.eclinm.2024.102703 PMC11261399

[7] Jani BD, Hanlon P, Nicholl BI, McQueenie R, Gallacher KI, Lee D, et al. Relationship between multimorbidity, demographic factors and mortality: findings from the UK Biobank cohort. BMC Med. 2019;17(1): 74. 30967141 10.1186/s12916-019-1305-x PMC6456941

[8] Storeng SH, Vinjerui KH, Sund ER, Krokstad S. Associations between complex multimorbidity, activities of daily living and mortality among older Norwegians. A prospective cohort study: the HUNT study, Norway. BMC Geriatr. 2020;20(1): 21. 31964341 10.1186/s12877-020-1425-3 PMC6974981

[9] Cassell A, Edwards D, Harshfield A, Rhodes K, Brimicombe J, Payne R, et al. The epidemiology of multimorbidity in primary care: a retrospective cohort study. Br J Gen Pract. 2018;68(669):e245–51. 29530918 10.3399/bjgp18X695465 PMC5863678

[10] Payne RA, Abel GA, Guthrie B, Mercer SW. The effect of physical multimorbidity, mental health conditions and socioeconomic deprivation on unplanned admissions to hospital: a retrospective cohort study. CMAJ. 2013;185(5):E221–8. 23422444 10.1503/cmaj.121349 PMC3602270

[11] Fortin M, Lapointe L, Hudon C, Vanasse A, Ntetu AL, Maltais D. Multimorbidity and quality of life in primary care: a systematic review. Health Qual Life Outcomes. 2004;2(1): 51. 15380021 10.1186/1477-7525-2-51 PMC526383

[12] Pan T, Anindya K, Devlin N, Mercer SW, McPake B, van Heusden A, et al. The impact of depression and physical multimorbidity on health-related quality of life in China: a national longitudinal quantile regression study. Sci Rep. 2022;12(1): 21620. 36517510 10.1038/s41598-022-25092-7 PMC9750988

[13] MacRae C, Mercer SW, Henderson D, McMinn M, Morales DR, Jefferson E, et al. Age, sex, and socioeconomic differences in multimorbidity measured in four ways: UK primary care cross-sectional analysis. Br J Gen Pract. 2023;73(729):e249–56. 36997222 10.3399/BJGP.2022.0405 PMC9923763

[14] May CR, Eton DT, Boehmer K, Gallacher K, Hunt K, Macdonald S, et al. Rethinking the patient: using burden of treatment theory to understand the changing dynamics of illness. BMC Health Serv Res. 2014;14: 281. 24969758 10.1186/1472-6963-14-281 PMC4080515

[15] Holland E, Matthews K, Macdonald S, Ashworth M, Laidlaw L, Cheung KSY, et al. The impact of living with multiple long-term conditions (multimorbidity) on everyday life – a qualitative evidence synthesis. BMC Public Health. 2024;24(1): 3446. 39696210 10.1186/s12889-024-20763-8 PMC11654051

[16] May C, Montori VM, Mair FS. We need minimally disruptive medicine. BMJ. 2009. 10.1136/bmj.b2803. 19671932 10.1136/bmj.b2803

[17] Shippee ND, Shah ND, May CR, Mair FS, Montori VM. Cumulative complexity: a functional, patient-centered model of patient complexity can improve research and practice. J Clin Epidemiol. 2012;65(10):1041–51. 22910536 10.1016/j.jclinepi.2012.05.005

[18] Rachele JN, Kavanagh AM, Badland H, Giles-Corti B, Washington S, Turrell G. Associations between individual socioeconomic position, neighbourhood disadvantage and transport mode: baseline results from the HABITAT multilevel study. J Epidemiol Community Health. 2015;69(12):1217–23. 26243197 10.1136/jech-2015-205620

[19] Hemmerechts K, Agirdag O, Kavadias D. The relationship between parental literacy involvement, socio-economic status and reading literacy. Educ Rev. 2017;69(1):85–101.

[20] Boehmer K, Gallacher K, Lippiett K, Mair F, May C, Montori V. Minimally disruptic medicine: progress 10 years later. Mayo Clin Proc. 2022;97(2):210–20. 35120690 10.1016/j.mayocp.2021.09.003

[21] Smyth RC, Smith G, Alexander E, May CR, Mair FS, Gallacher KI. A systematic review of the use of burden of treatment theory. J Multimorb Comorb. 2025;15: 26335565251314828. 40352785 10.1177/26335565251314828 PMC12064904

[22] Gallacher K, May C, Montori VM, Mair FS. Understanding treatment burden in chronic heart failure patients: a qualitative study. Ann Fam Med. 2011;9. 10.1370/afm.1249 PMC3090432 21555751

[23] Gallacher K, Morrison D, Jani B, Macdonald S, May CR, Montori VM, et al. Uncovering treatment burden as a key concept for stroke care: a systematic review of qualitative research. PLoS Med. 2013;10(6): e1001473. 23824703 10.1371/journal.pmed.1001473 PMC3692487

[24] Kyle J, Skleparis D, Mair FS, Gallacher KI. What helps and hinders the provision of healthcare that minimises treatment burden and maximises patient capacity? A qualitative study of stroke health professional perspectives. BMJ Open. 2020;10(3): e034113. 32193265 10.1136/bmjopen-2019-034113 PMC7150601

[25] Adam R, Nair R, Duncan LF, Yeoh E, Chan J, Vilenskaya V, et al. Treatment burden in individuals living with and beyond cancer: a systematic review of qualitative literature. PLoS One. 2023;18(5): e0286308. 37228101 10.1371/journal.pone.0286308 PMC10212163

[26] Chikumbu EF, Bunn C, Kasenda S, Dube A, Phiri-Makwakwa E, Jani BD, et al. Experiences of multimorbidity in urban and rural Malawi: an interview study of burdens of treatment and lack of treatment. PLoS Glob Public Health. 2022;2(3): e0000139. 36962280 10.1371/journal.pgph.0000139 PMC10021162

[27] van Pinxteren M, Mbokazi N, Murphy K, Mair FS, May C, Levitt N. The impact of persistent precarity on patients’ capacity to manage their treatment burden: a comparative qualitative study between urban and rural patients with multimorbidity in South Africa. Front Med. 2023. 10.3389/fmed.2023.1061190. 10.3389/fmed.2023.1061190 PMC10098191 37064034

[28] van Pinxteren M, Mbokazi N, Murphy K, Mair FS, May C, Levitt NS. Using qualitative study designs to understand treatment burden and capacity for self-care among patients with HIV/NCD multimorbidity in South Africa: a methods paper. J Multimorb Comorb. 2023;13: 26335565231168041. 37057034 10.1177/26335565231168041 PMC10088413

[29] Mbokazi N, van Pinxteren M, Murphy K, Mair FS, May CR, Levitt NS. Ubuntu as a mediator in coping with multimorbidity treatment burden in a disadvantaged rural and urban setting in South Africa. Soc Sci Med. 2023;334: 116190. 37659263 10.1016/j.socscimed.2023.116190

[30] Tarzia L, May C, Hegarty K. Assessing the feasibility of a web-based domestic violence intervention using chronic disease frameworks: reducing the burden of ‘treatment’ and promoting capacity for action in women abused by a partner. BMC Womens Health. 2016;16(1): 73. 27881163 10.1186/s12905-016-0352-0 PMC5122198

[31] Duncan P, Murphy M, Man M-S, Chaplin K, Gaunt D, Salisbury C. Development and validation of the multimorbidity treatment burden questionnaire (MTBQ). BMJ Open. 2020;8(4): e019413. 10.1136/bmjopen-2017-019413 PMC5900423 29654011

[32] Eton DT, Linzer M, Boehm DH, Vanderboom CE, Rogers EA, Frost MH, et al. Deriving and validating a brief measure of treatment burden to assess person-centered healthcare quality in primary care: a multi-method study. BMC Fam Pract. 2020;21(1): 221. 33115421 10.1186/s12875-020-01291-x PMC7594460

[33] Tran V-T, Harrington M, Montori VM, Barnes C, Wicks P, Ravaud P. Adaptation and validation of the treatment burden questionnaire (TBQ) in English using an internet platform. BMC Med. 2014;12(1):109. 24989988 10.1186/1741-7015-12-109 PMC4098922

[34] Tran VT, Montori VM, Ravaud P. Is my patient overwhelmed? Determining thresholds for acceptable burden of treatment using data from the ComPaRe e-cohort. Mayo Clin Proc. 2020;95(3):504–12. 31619365 10.1016/j.mayocp.2019.09.004

[35] Sav A, Whitty JA, McMillan SS, Kendall E, Kelly F, King MA, et al. Treatment burden and chronic illness: who is at most risk? Patient. 2016;9(6):559–69. 27142372 10.1007/s40271-016-0175-y

[36] Jones C, Mair FS, Williamson AE, McPherson A, Eton DT, Lowrie R. Treatment burden for people experiencing homelessness with a recent non-fatal overdose: a questionnaire study. Br J Gen Pract. 2023;73(735):e728–34. 37429734 10.3399/BJGP.2022.0587 PMC10355813

[37] Macintyre S, Ellaway A, Der G, Ford G, Hunt K. Do housing tenure and car access predict health because they are simply markers of income or self esteem? A Scottish study. J Epidemiol Community Health. 1998;52(10):657–64. 10023466 10.1136/jech.52.10.657 PMC1756620

[38] Reeves D, Blickem C, Vassilev I, Brooks H, Kennedy A, Richardson G, et al. The contribution of social networks to the health and self-management of patients with long-term conditions: a longitudinal study. PLoS One. 2014;9(6): e98340. 24887107 10.1371/journal.pone.0098340 PMC4041782

[39] Koetsenruijter J, van Eikelenboom N, van Lieshout J, Vassilev I, Lionis C, Todorova E, et al. Social support and self-management capabilities in diabetes patients: an international observational study. Patient Educ Couns. 2016;99(4):638–43. 26549171 10.1016/j.pec.2015.10.029

[40] Fan Z-y, Yang Y, Zhang F. Association between health literacy and mortality: a systematic review and meta-analysis. Arch Public Health. 2021;79(1): 119. 34210353 10.1186/s13690-021-00648-7 PMC8247180

[41] Vassilev I, Rogers A, Kennedy A, Koetsenruijter J. The influence of social networks on self-management support: a metasynthesis. BMC Public Health. 2014;14: 719. 25023948 10.1186/1471-2458-14-719 PMC4223639

[42] Vassilev I, Rogers A, Kennedy A, Wensing M, Koetsenruijter J, Orlando R, et al. Social network type and long-term condition management support: a cross-sectional study in six European countries. PLoS One. 2016;11(8): e0161027. 27536988 10.1371/journal.pone.0161027 PMC4990169

[43] Lund Jensen AQ, Pedersen HS, Vestergaard M, Mercer SW, Glümer C, Prior A. The impact of socioeconomic status and multimorbidity on mortality: a population-based cohort study. Clin Epidemiol. 2017;9(null):279–89. 28546772 10.2147/CLEP.S129415 PMC5436773

[44] Nunes BP, Flores TR, Mielke GI, Thumé E, Facchini LA. Multimorbidity and mortality in older adults: a systematic review and meta-analysis. Arch Gerontol Geriatr. 2016;67:130–8. 27500661 10.1016/j.archger.2016.07.008

[45] St John PD, Tyas SL, Menec V, Tate R. Multimorbidity, disability, and mortality in community-dwelling older adults. Can Fam Physician. 2014;60(5):e272–80. 24829022 PMC4020665

[46] Macintyre S, Ellaway A, Cummins S. Place effects on health: how can we conceptualise, operationalise, and measure them? Soc Sci Med. 2002;55(1):125–39. 12137182 10.1016/s0277-9536(01)00214-3

[47] Smith KE, Anderson R. Understanding lay perspectives on socioeconomic health inequalities in Britain: a meta-ethnography. Sociol Health Illn. 2018;40(1):146–70. 29044572 10.1111/1467-9566.12629

[48] Zheng C, MacRae C, Rowley-Abel L, Arakelyan S, Abubakar E, Dibben C, et al. The impact of place on multimorbidity: a systematic scoping review. Soc Sci Med. 2024;361: 117379. 39447514 10.1016/j.socscimed.2024.117379

[49] Pathirana TI, Jackson CA. Socioeconomic status and multimorbidity: a systematic review and meta-analysis. Aust N Z J Public Health. 2018;42(2):186–94. 29442409 10.1111/1753-6405.12762

[50] Ingram E, Ledden S, Beardon S, Gomes M, Hogarth S, McDonald H, et al. Household and area-level social determinants of multimorbidity: a systematic review. J Epidemiol Community Health. 2021;75(3):232–41. 33158940 10.1136/jech-2020-214691 PMC7892392

[51] Benzeval M, Der G, Ellaway A, Hunt K, Sweeting H, West P, et al. Cohort profile: west of Scotland twenty-07 study: health in the community. Int J Epidemiol. 2009;38(5):1215–23. 18930962 10.1093/ije/dyn213 PMC2935558

[52] Der GA. A comparison of the West of Scotland Twenty-07 studysample and the 1991 Census SARs. Working Paper No. 60. Glasgow: MRC Medical Sociology Unit; 1998.

[53] Katikireddi SV, Skivington K, Leyland AH, Hunt K, Mercer SW. The contribution of risk factors to socioeconomic inequalities in multimorbidity across the lifecourse: a longitudinal analysis of the twenty-07 cohort. BMC Med. 2017;15(1): 152. 28835246 10.1186/s12916-017-0913-6 PMC5569487

[54] MRC Social and Public Health Sciences Unit. West of Scotlan Twenty -07 study health in the community [cited 2022 23 May]. Available from: http://2007study.sphsu.mrc.ac.uk/.

[55] Brown D AM, Dundas R, Leyland A. Carstairs deprivation score for Scotland 2022 [updated 8 April 2022; cited 2022 24 May]. Available from: https://researchdata.gla.ac.uk/1270/.

[56] Janssen KJM, Donders ART, Harrell FE Jr., Vergouwe Y, Chen Q, Grobbee DE, et al. Missing covariate data in medical research: to impute is better than to ignore. J Clin Epidemiol. 2010;63(7):721–7. 20338724 10.1016/j.jclinepi.2009.12.008

[57] Buckton CH, Lean MEJ, Combet E. ‘Language is the source of misunderstandings’–impact of terminology on public perceptions of health promotion messages. BMC Public Health. 2015;15(1): 579. 26100790 10.1186/s12889-015-1884-1 PMC4476206

[58] Henninger DE, Whitson HE, Cohen HJ, Ariely D. Higher medical morbidity burden is associated with external locus of control. J Am Geriatr Soc. 2012;60(4):751–5. 22458257 10.1111/j.1532-5415.2012.03904.x PMC3325320

[59] van den Akker M, Buntinx F, Metsemakers JFM, van der Aa M, Knottnerus JA. Psychosocial patient characteristics and GP-registered chronic morbidity: a prospective study. J Psychosom Res. 2001;50(2):95–102. 11274666 10.1016/s0022-3999(00)00227-0

[60] van der Linden M, van den Akker M, Buntinx F. The relation between health locus of control and multimorbidity: a case-control study. Pers Individ Dif. 2001;30(7):1189–97.

[61] Mounce LTA, Campbell JL, Henley WE, Tejerina Arreal MC, Porter I, Valderas JM. Predicting incident multimorbidity. Ann Fam Med. 2018;16(4):322–9. 29987080 10.1370/afm.2271 PMC6037507

[62] Olaya B, Domènech-Abella J, Moneta MV, Lara E, Caballero FF, Rico-Uribe LA, et al. All-cause mortality and multimorbidity in older adults: the role of social support and loneliness. Exp Gerontol. 2017;99:120–6. 28982608 10.1016/j.exger.2017.10.001

[63] Schäfer I, Hansen H, Kaduszkiewicz H, Bickel H, Fuchs A, Gensichen J, et al. Health behaviour, social support, socio-economic status and the 5-year progression of multimorbidity: results from the multicare cohort study. J Comorb. 2019;9: 2235042X19883560. 35174099 10.1177/2235042X19883560 PMC8842469

[64] Lozano-Hernández CM, López-Rodríguez JA, Leiva-Fernández F, Calderón-Larrañaga A, Barrio-Cortes J, Gimeno-Feliu LA, et al. Social support, social context and nonadherence to treatment in young senior patients with multimorbidity and polypharmacy followed-up in primary care. MULTIPAP study. PLoS One. 2020;15(6): e0235148. 32579616 10.1371/journal.pone.0235148 PMC7314051

[65] Hanlon P, Nicholl BI, Jani BD, Lee D, McQueenie R, Mair FS. Frailty and pre-frailty in middle-aged and older adults and its association with multimorbidity and mortality: a prospective analysis of 493 737 UK Biobank participants. Lancet Public Health. 2018;3(7):e323–32. 29908859 10.1016/S2468-2667(18)30091-4 PMC6028743

[66] Vetrano DL, Palmer K, Marengoni A, Marzetti E, Lattanzio F, Roller-Wirnsberger R, et al. Frailty and multimorbidity: a systematic review and meta-analysis. J Gerontol A Biol Sci Med Sci. 2018;74(5):659–66. 10.1093/gerona/gly110 29726918

[67] Villacampa-Fernández P, Navarro-Pardo E, Tarín JJ, Cano A. Frailty and multimorbidity: two related yet different concepts. Maturitas. 2017;95:31–5. 27889050 10.1016/j.maturitas.2016.10.008

[68] Hibbard JH, Stockard J, Mahoney ER, Tusler M. Development of the Patient Activation Measure (PAM): conceptualizing and measuring activation in patients and consumers. Health Serv Res. 2004;39(4 Pt 1):1005–26. 15230939 10.1111/j.1475-6773.2004.00269.x PMC1361049

[69] VanderWeele TJ. On the promotion of human flourishing. Proc Natl Acad Sci U S A. 2017;114(31):8148–56. 28705870 10.1073/pnas.1702996114 PMC5547610

