# Supplementary material for: Measuring individual and community capacity factors in people with multimorbidity and exploring associations with health outcomes
Source: BMC Med. 2025 Oct 16;23:566. doi: 10.1186/s12916-025-04337-y (PMC12532429; doi:10.1186/s12916-025-04337-y)
Supplement: Supplementary file 1 — Supplementary material 1. Figure S1: Mobilising Capacity DAG. Figure S2: Expressing Capacity DAG. Figure S3: Community Capacity DAG. Table S1: Conditions used to identify Multimorbidity. Table S2: Mobilising Capacity variables. Table S3: Expressing Capacity Variable. Table S4: community Capacity Variables. Table S5: Table demonstrating Hazard Ratio for Mobilising Capacity Variables when not adjustingor adjustingfor presence of absence of multimorbidity. Table S6: Table demonstrating Odds Ratio for self-reported hospital admission for Mobilising Capacity Variables when not adjustingor adjustingfor presence of absence of multimorbidity. Table S7: Table demonstrating Hazard Ratio for Expressing Capacity Variables when not adjustingor adjustingfor presence of absence of multimorbidity. Table S8: Table demonstrating Odds Ratio for self-reported hospital admission for Expressing Capacity Variables when not adjustingor adjustingfor presence of absence of multimorbidity. Table S9: demonstrating Hazard Ratio for Community Capacity Variables when not adjustingor adjustingfor presence of absence of multimorbidity. Table S10 demonstrating Odds Ratio for self-reported hospital admission for Mobilising Capacity Variables when not adjustingor adjustingfor presence of absence of multimorbidity. [file 12916_2025_4337_MOESM1_ESM.docx]

**Supplementary Information**

**Supplementary tables S1: Conditions used to identify Multimorbidity…………….2**

**2- S4: Summary of how each variable was measured, and any issues with missingness, for each construct…………………………………………….2**

**Supplementary Figures S1-S3: DAGs exploring the impact of variables and potential confounders on mortality utilising potential variables from the WoS 20-07 cohort……………………………………………………………………………………………………………..9**

**Tables S5-S10: Sensitivity Analysis using multimorbidity as a time varying covariate………………………………………………………………………………………………………………….11**

**Table S1:Conditions used to identify Multimorbidity**

Previous coding of multimorbidity was done prior to this project and written up in a previous paper, including this table summarising their work(48). The conditions used to classify Multimorbidity, and their definitions were based on coding of multimorbidity on the basis of the Royal College of General Practitioners’ 1986 classification system:

| **CONDITION** | **CODE** |
| --- | --- |
| **HYPERTENSION** | 1910* |
|  | 1915* |
|  | 1920* |
|  | 1925* |
|  | 1935* |
| **DEPRESSION** | 1060 |
| **RESPIRATORY CONDITIONS including asthma** | 2500 |
|  | 2510 |
|  | 2490* |
|  | 2495* |
| **CORONARY HEART DISEASE** | 1940* |
|  | 1945* |
|  | 1950* |
|  | 7735* |
| **DYSPEPSIA** | 2660 |
|  | 2675 |
|  | 2680 |
|  | 2685 |
|  | 2690 |
|  | 2695 |
|  | 2700 |
|  | 4660 |
| **DIABETES** | 0720* |
| **THYROID** | 0700 |
|  | 0705* |
|  | 0710* |
|  | 0715* |
|  | 7570* |
| **RHEUMATOID ARTHRITIS, other inflammatory polyarthropathies & systematic connective tissue disorders** | 3905* |
|  | 3910* |
|  | 4510* |
|  | 0770 |
|  | 3690* |
|  | 3900* |
| **HEARING PROBLEMS** | 1740 |
|  | 1745 |
|  | 1750 |
|  | 1770 |
|  | 1775 |
|  | 1780 |
| **ANXIETY & OTHER NEUROTIC, STRESS-RELATED, & SOMATOFORM DISORDERS** | 1040 |
|  | 1045 |
|  | 1050 |
|  | 1055 |
|  | 1150 |
|  | 1155 |
|  | 1160 |
|  | 1175 |
| **IBS** | 2770 |
| **CANCER** | 0400 TO 0590 |
| **ALCOHOL PROBLEMS** | 1005* |
|  | 1100* |
|  | 1115 |
| **OTHER PSYCHOACTIVE SUBSTANCE MISUSE** | 1010 |
|  | 1110 |
| **CONSTIPATION** | 2765 |
| **STROKE & TRANSIENT ISCHAEMIC ATTACK** | 2100* |
|  | 2105* |
|  | 2110* |
|  | 2115* |
| **CHRONIC KIDNEY DISEASE** | 2900* |
|  | 2905* |
| **DIVERTICULAR DISEASE OF INTESTINE** | 2760* |
| **ATRIAL FIBRILLATION** | 1990* |
| **PERIPHERAL VASCULAR DISEASE** | 2200* |
|  | 2210 |
|  | 2215* |
|  | 2220* |
|  | 2225* |
|  | 2230* |
| **HEART FAILURE** | 2005* |
|  | 2010* |
|  | 2015* |
|  | 2020* |
| **PROSTATE DISORDERS** | 2960 |
|  | 2970 |
|  | 7960 |
|  | 7965 |
| **GLAUCOMA** | 1535* |
| **EPILEPSY** | 1365 |
|  | 1370 |
|  | 1375 |
| **DEMENTIA** | 1000* |
|  | 1310* |
|  | 4900* |
| **PSYCHOSES** | 1020* |
|  | 1025* |
|  | 1030* |
| **PSORIASIS OR ECZEMA** | 3695 |
|  | 3655 |
|  | 3665 |
| **INFLAMMATORY BOWEL DISEASE** | 2740* |
|  | 2745* |
| **MIGRAINE** | 1380 |
| **BLINDNESS & LOW VISION** | 1555* |
| **CHRONIC SINUSITIS** | 2445 |
| **LEARNING DISABILITY** | 1215* |
|  | 1220* |
| **ANOREXIA OR BULIMIA** | 1170* |
|  | 1180* |
| **BRONCHIECTASIS** | 2505* |
| **PARKINSON’S DISEASE** | 1315* |
|  | 1320* |
| **MULTIPLE SCLEROSIS** | 1335* |
| **VIRAL HEPATITIS** | 0130 |
| **CHRONIC LIVER DISEASE** | 2810* |
|  | 4560* |
| **PAIN** | 1190 |
|  | 3910 |
|  | 3915 |
|  | 3920 |
|  | 3925 |
|  | 3930 |
|  | 3935 |
|  | 3940 |
|  | 3945 |
|  | 3950 |
|  | 3955 |
|  | 3960 |
|  | 3980 |
|  | 3990 |
|  | 3995 |
|  | 4000 |
|  | 4005 |
|  | 4075 |
|  | 4080 |

**Summary** **of how each variable was measured, and any issues with missingness, for each construct**

**Table S2: Mobilising Capacity variables**

| **Variable** | **How Measured** | **Responses** | **Missing** |
| --- | --- | --- | --- |
| Income | Participant asked to estimate household income, result then equivalised and adjusted to 1987 inflation to allow comparison across waves | Numerical | Asked across all waves and cohorts |
| Car access | Do you, or your household own a car or van? | Yes/no response | Asked across all waves and cohorts |
| Housing Tenancy | Participant asked to identify housing tenancy type | “Owner”, “Social Housing”, “private rental”, “other tenure” | Asked across all waves and cohorts |
| Employment status | Participants requested to self-identify employment status | Full time education, employed/self-employed, carer or housewife, retired, unemployed, disabled. | Asked across all waves and cohorts |
| Life event – death family member | Combination value created for people who replied affirmatively to death of spouse, child, or close member of family in the previous two years | Yes/No | Questions asked differently across the waves. Cohort two at baseline were asked separate questions about “life events” in general, which created a score with no information on individual events. The youngest cohort was asked slightly different questions in waves 1-2, and there was no information on employment changes for this cohort. Wave three locality sample not directly asked about employment changes. |
| Life event -death friend | Death of a close friend in the last two years | Yes/No |  |
| Life event – divorce | Divorce in the last two years | Yes/No |  |
| Life events -unemployment | Been made unemployed in the last two years | Yes/No |  |
| Life events – job change | Significant change in job status in the last two years | Yes/No |  |
| Number family contacts/month | Wave 1-3 recorded number of times were in contact with different family members in last 4 weeks, waves 4 and 5 only record if family member were seen in last four weeks. Binary variable therefore used in analysis to allow comparison across waves | Yes/No | Asked across all waves and cohorts but different questions across the waves. |
| Number friend contacts/month | Wave 1-3 recorded number of times were in contact with friends in last 4 weeks, waves 4 and 5 only record if family member were seen in last four weeks. Binary variable used in analysis to allow comparison across waves | Yes/No | Asked across all waves and cohorts but different questions across the waves. |
| Share feelings | Have you someone you can share your feelings with? | “All feelings”, “some”, “few” | W1 only asked oldest cohort, W2 only asked oldest and middle aged, W3 only asked regional sample. |
| Someone to confide in | Have you someone you can confide in? | “Very Frequently”, “quite often”, “Occasionally”, “Never” | W1 only asked oldest cohort, W2 only asked oldest and middle aged, W3 only asked regional sample. |
| Someone to offer practical Support | About how many people could you ask for practical help? | Numerical | Not asked W1, only oldest and middle-aged cohort W2, W3 only asked regional sample. |
| Loneliness | Do you ever feel lonely? | Never, seldom, occasionally, quite often, most of the time | W1 only asked oldest cohort, W2 only asked middle aged and oldest cohorts. |
| Carer | Are you a carer? | Yes/No | W1 only asked oldest cohort, W3 only asked regional sample |

**Table S3: Expressing Capacity Variables**

| Variable | How Measured | Responses | Missing |
| --- | --- | --- | --- |
| Self esteem | Measured using Rosenberg’s Self-Esteem Score: numerical score ranging from 10(low) to 40 (high)[1] | Numerical score | Only asked in waves 2, 3 and 4 (only regional sample in wave 3). As felt to be of particular importance in expressing capacity included. |
| Self-reported Health | Over the last 12 months would you say your health on the whole has been…… | Good, Fairly good, Not good | Asked across all waves and cohorts |
| Health compared to Others your age | Would you say that for someone your own age your health in general is… | Excellent, Good, Fair, Poor | W1 only asked oldest cohort, W2 only asked oldest and middle aged, W3 only asked regional sample. |
| Health Locus of control | Set of statements:   - I have the power to make myself well - I have no control over being ill - Regular doctor visits reduce health - Accidental happening influence health - Only doctors can maintain health - I am responsible for my health - Others are responsible for my health - It’s my fault when things go wrong with my health - When I am ill, I let nature run its course - When I’m health it’s because I am lucky - Wellbeing depends on taking care of yourself - Illness means you have not cared for yourself - Care from others helps me to get well - Illness is luck - Looking after myself keeps me healthy - Doctor’s orders keep me healthy | Respondents could agree strongly, agree quite a bit, agree a little, disagree a little, disagree quite a bit, disagree strongly | All cohorts asked in W2 only. ). As felt to be of particular importance in expressing capacity included. |
| Mastery | Set of statements:   - I have little control over what happens to me - There is no way I can solve some of the problems I have - There is little I can do to change many of the important things in my life - Sometimes I feel helpless dealing with the problems in life - Sometimes I feel I am pushed around in life - What happens in the future depends mostly on me - I can do just about anything I set my mind to | Strongly agree, agree, disagree, strongly disagree | All cohorts asked in wave 4 only. ). As felt to be of particular importance in expressing capacity included. |
| Disability | Are you registered as disabled? | Yes/No | Not asked wave 1, otherwise asked all cohorts each wave |
| Life limiting LTC | All participants were asked if their LTCs had a limit on their life | Not limited by LTC, limited by LTC | Asked across all cohorts and waves |
| Anxiety | Hospital Anxiety and Depression questionnaire (HADS), scores categorised by clinical cut-offs | No, mild, moderate, or severe anxiety | Asked across all cohorts and waves |
| Depression | HADS questionnaire scores categorised by clinical cut-offs | No, mild, moderate, or severe depression | Asked across all cohorts and waves |
| Educational attainment by age 35 | Max educational attainment by age 35 (oldest age of youngest cohort at final wave) was used as a measure of someone’s **potential** educational achievement as a single variable, across the cohort (the alternative would have been to look at educational achievement at each wave but as anyone from the youngest cohort was only 15 years at baseline this would have introduced significant bias). | Apprenticeship, Standard Grade, Higher, HND, degree. | One off variable summarising maximum educational achievement by the age of 35 (oldest age of the youngest group when the cohort finished). This allowed measure of educational attainment that could be standardised across the three cohorts. |
| Alice Heim 4 Test (AH4) | Standardised assessment, questions answered within a specific time that measures verbal and non-verbal ability | Numerical | Measured oldest cohort W1, and all three cohorts W4 and W5. As would be expected very high correlation between the three scores. |
| Number of community Groups | Number of community groups involved in | Numerical | W1 only asked oldest cohort, W2 only asked oldest and middle aged, W3 only asked regional sample. |

**Table S4: community Capacity Variables**

| Variable | How Measured | Responses | Missingness |
| --- | --- | --- | --- |
| How do you feel about your area? | Looking at the faces scale which face shows best how you feel about living in the area? | Faces identified on scale 1-7 with 1 most satisfied | Only asked oldest cohort W1, and oldest and middle-aged cohorts W2 |
| Neighbourliness | Do you exchange small favours with the people who live near? I am thinking about things like leaving a key to let a repair man in? | Yes/No | Only asked oldest cohort W1, and oldest and middle-aged cohorts W2, only asked regional sample W3 |
| Walking in the dark | How do you feel about walking around the area after dark? Would you say that you… | Never do it in any circumstances, try to avoid it, if possible, do it but feel uncomfortable, have no worries about doing it | Only asked oldest cohort W1, and oldest and middle-aged cohorts W2 |
| Problem – vandalism | Around where you live would you say vandalism is a problem? | Serious problem, a minor problem, not a problem | Only asked oldest cohort W1, and oldest and middle-aged cohorts W2 |
| Problem – litter | Around where you live would you say litter is a problem? | Serious problem, a minor problem, not a problem | Only asked oldest cohort W1, and oldest and middle-aged cohorts W2 |
| Problem – assaults/ muggings | Around where you live would you say assaults/muggings are a problem? | Serious problem, a minor problem, not a problem | Only asked oldest cohort W1, and oldest and middle-aged cohorts W2 |
| Problem – burglaries | Around where you live would you say burglaries are a problem? | Serious problem, a minor problem, not a problem | Only asked oldest cohort W1, and oldest and middle-aged cohorts W2 |
| Problem – young people causing disturbance | Around where you live would you say young people causing disturbance is a problem? | Serious problem, a minor problem, not a problem | Only asked oldest cohort W1, and oldest and middle-aged cohorts W2 |

**DAG s exploring the impact of vairables and potential confounders on mortality utilising potential variables from the WoS 20-07 cohort**^[[1]](#footnote-1)^

**Figure S1: Mobilising Capacity DAG**


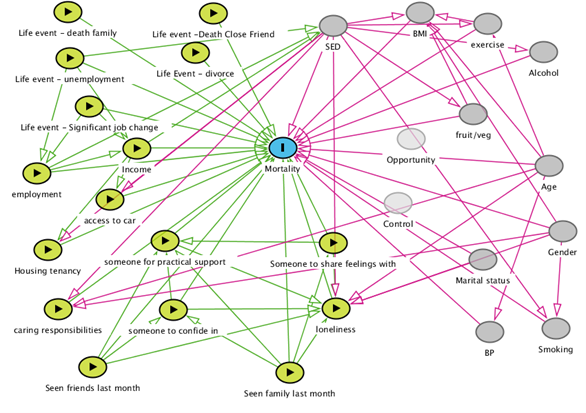


Figure S2: Expressing Capacity DAG


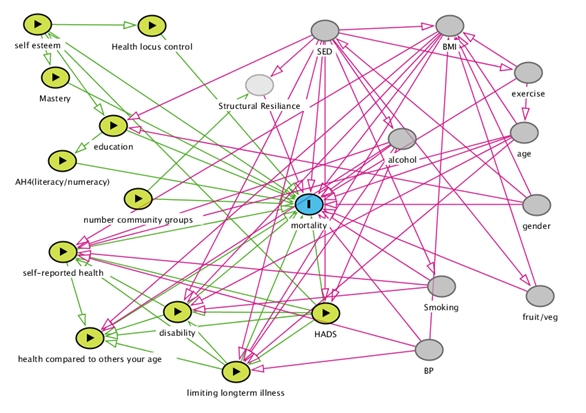


Figure S3: Community Capacity DAG


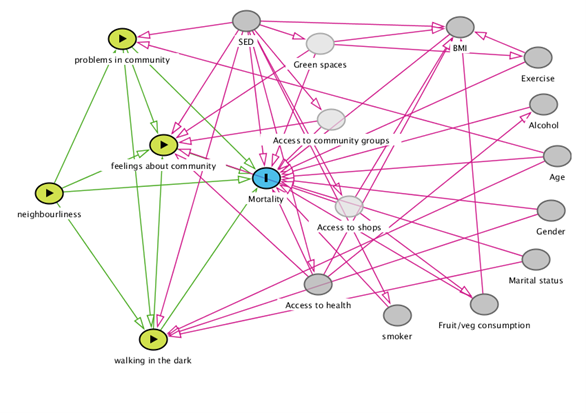


**Sensitivity Analysis using multimorbidity as a time varying covariate**

The analysis was done on a subset of the original cohort who had multimorbidity at any of the waves within the cohort. This meant at each wave there were a small number whose may not have had multimorbidity at a particular wave (because they had not developed it, or because of the self-reported nature of the variable, discussed in Chapter 9). To explore whether this influenced the findings a sensitivity analysis was conducted:

For the mortality tables:

- Model 1 is the Hazard Ratio fully adjusted for the existing confounders presented in Chapter 8
- Model 2 is the Hazard Ratio for model 1 where presence or absence of multimorbidity has been included as a time-varying covariate.

For the Hospital admission tables:

- Model 1 is the Odds Ratio fully adjusted for the existing confounders presented in Chapter 8
- Model 2 is the Odds Ratio adjusting for all the existing confounders and presence or absence of multimorbidity.

The Sensitivity analysis showed very little change in effect, or confidence interval size.

**Table S5: Table demonstrating Hazard Ratio for Mobilising Capacity Variables when not adjusting (Model 1) or adjusting (Model 2) for presence of absence of multimorbidity.**

| Variables | Hazard Ratio (Confidence Interval) | |
| --- | --- | --- |
|  | Model 1 | Model 2 |
| Equivalised Household Income | 1 (1,1) | 1 (1,1) |
| Housing Tenancy  Owner  Social Housing  Private Rent  Other | 1  1.39 (1.14,1.68)**  1.23 (0.7,2.17)  1.28 (0.62,2.64) | 1  1.39 (1.14,1.68)**  1.18 (0.67,2.09)  1.41 (0.68,2.9) |
| Access to a car  Yes  No | 1  1.13 (0.93,1.36)) | 1  1.13 (0.94,1.37) |
| Employment Status  Employed/self-employed  Full Time Education  Carer/Housewife  Retired  Unemployed  Disabled | 1  3.70 (0.83,16.52)  2.00 (1.26,3.19)**  1.63 (1.14,2.33)**  1.83 (0.97,3.44)  2.88 (1.98,4.19)** | 1  3.47 (0.78,15.48)  1.96 (1.23,3.13)  1.60 (1.12,2.3)  1.74 (0.92,3.29)  2.68 (1.84,3.91) |
| Seen Family member in the last month?  Yes  No | 1  1.31 (0.54,3.17) | 1  1.31 (0.54,3.19) |
| Seen Friend in the last month?  Yes  No | 1  0.61 (0.25,1.49) | 1  0.62 (0.25,1.52) |
| Number of people you can rely on for practical support  None  Up to five  Five to ten  More than ten | 1  1.04 (0.76,1.43)  1.03 (0.71,1.50)  1.07 (0.62,1.87) | 1  1.02 (0.74,1.4)  1.00 (0.69,1.45)  1.05 (0.61,1.83) |
| Do you ever feel lonely?  Never  Seldom  Occasionally  Quite often  Most of the time | 1  0.68 (0.51,0.91)**  0.89 (0.71,1.12)  1.74 (1.27,2.39)**  0.93 (0.58,1.49) | 1  0.68 (0.51,0.92)**  0.88 (0.70,1.10)  1.66 (1.21,2.28)**  0.91 (0.57,1.45) |
| Have you someone you can share your feelings with?  All  Some feelings  A few feelings | 1  0.79 (0.64,0.99)*  0.68 (0.48,0.95)* | 1  0.81 (0.65,1.01)  0.68 (0.49,0.96)* |
| Have you someone you can confide in?  Very frequently  More often than not  Occasionally  Never | 1  1.12 (0.75,1.68)  1.17 (0.80,1.70)  1.02 (0.71,1.47) | 1  1.13 (0.75,1.69)  1.18 (0.81,1.72)  1.04 (0.72,1.51) |
| Are you a carer?  Yes  No | 1  0.72 (0.58,0.90)* | 1  0.72 (0.58,0.90)* |
| Divorced in the last year?  Yes  No | 1  2.07 (1.11,3.87) | 1  2.13 (1.14,3.98)* |
| Become unemployed in the last year?  Yes  No | 1  0.71 (0.30,1.70) | 1  0.71 (0.30,1.68) |
| Change in employment in the last year?  Yes  No | 1  1.2 (0.59,2.46) | 1  1.18 (0.58,2.41)) |
| Death in the family  No  Yes | 1  0.79 (0.62,1.00) | 1  0.79 (0.63,1.00) |
| Death of friend in the last year?  No  Yes | 1  0.89 (0.71,1.12) | 1  0.88 (0.7,1.1) |

**Table S6: Table demonstrating Odds Ratio for self-reported hospital admission for Mobilising Capacity Variables when not adjusting (Model 1) or adjusting (Model 2) for presence of absence of multimorbidity.**

| Variables | Odds Ratio for self-reported hospital admission in the last year (95% Confidence Interval) | |
| --- | --- | --- |
|  | Model 1 | Model 2 |
| Equivalised Household Income | 1 (1,1) | 1 (1,1) |
| Housing Tenancy  Owner  Social Housing  Private Rent  Other | 1  1.2 (0.89,1.61)  1.3 (0.58,2.64)  1.61 (0.44,4.63) | 1  1.19 (0.88,1.6)  1.29 (0.58,2.62)  1.52 (0.42,4.39) |
| Access to a car  Yes  No | 1  1.00 (0.75,1.32) | 1  1.01 (0.76,1.34) |
| Employment Status  Employed/self-employed  Full Time Education  Carer/Housewife  Retired  Unemployed  Disabled | 1  0.55 (0.03,3.02)  1.74 (1.07,2.78)  2.43 (1.61,3.69)**  1.12 (0.5,2.31)  2.57 (1.67,3.95)** | 1  0.52 (0.03,2.9)  1.69 (1.04,2.7)  2.38 (1.58,3.61)**  1.08 (0.48,2.23)  2.44 (1.57,3.75)** |
| Seen Family member in the last month?  Yes  No | 1  0.74 (0.42,1.41) | 1  0.72 (0.41,1.37) |
| Seen Friend in the last month?  Yes  No | 1  1.14 (0.6,2.04) | 1  1.19 (0.62,2.13) |
| Number of people you can rely on for practical support  None  Up to five  Five to ten  More than ten | 1  1.50 (0.88,2.72)  1.74 (0.96,3.28)  1.60 (0.69,3.64) | 1  1.47 (0.86,2.66)  1.68 (0.93,3.17)  1.57 (0.68,3.57) |
| Do you ever feel lonely?  Never  Seldom  Occasionally  Quite often  Most of the time | 1  0.93 (0.62,1.35)  0.76 (0.54,1.05)  1.15 (0.72,1.8)  1.31 (0.61,2.68) | 1  0.92 (0.62,1.34)  0.75 (0.54,1.05)  1.11 (0.69,1.75)  1.26 (0.59,2.57) |
| Have you someone you can share your feelings with?  All  Some feelings  A few feelings | 1  0.85 (0.61,1.16)  0.62 (0.32,1.1) | 1  0.85 (0.62,1.16)  0.62 (0.32,1.11) |
| Have you someone you can confide in?  Very frequently  More often than not  Occasionally  Never | 1  1.03 (0.57,1.9)  0.72 (0.42,1.27)  0.8 (0.47,1.39) | 1  1.03 (0.57,1.91)  0.72 (0.42,1.27)  0.81 (0.48,1.41) |
| Are you a carer?  Yes  No | 1  0.75 (0.56,0.99) | 1  0.76 (0.57,1) |
| Divorced in the last year?  Yes  No | 1  1.59 (0.64,3.78) | 1  1.52 (0.61,3.63) |
| Become unemployed in the last year?  Yes  No | 1  1.05 (0.34,2.72) | 1  1.02 (0.33,2.64) |
| Change in employment in the last year?  Yes  No | 1  0.52 (0.2,1.15) | 1  0.51 (0.19,1.13) |
| Death in the family  No  Yes | 1  0.89 (0.59,1.32) | 1  0.89 (0.59,1.3) |
| Death of friend in the last year?  No  Yes | 1  1.32 (0.92,1.85) | 1  1.26 (0.88,1.78) |

**Table S7: Table demonstrating Hazard Ratio for Expressing Capacity Variables when not adjusting (Model 1) or adjusting (Model 2) for presence of absence of multimorbidity.**

| Variables | Hazard Ratio  (95% Confidence interval) | |
| --- | --- | --- |
|  | Model 1 | Model 2 |
| Over the last 12 months would you say your health on the whole has been……  Good  Fairly good  Not good | 1  1.14 (0.84,1.55)  1.19 (0.77,1.83) | 1  1.12 (0.82,1.51)  1.16 (0.75,1.78) |
| Would you say that for someone your own age your health in general is…  Excellent  Good  Fair  Poor | 1  0.94 (0.65,1.36)  1.38 (0.89,2.16)  2.18 (1.17,4.04)** | 1  0.94 (0.65,1.36)  1.38 (0.88,2.16)  2.15 (1.16,4)** |
| Registered disability  No  Yes | 1  1.7 (1.28,2.27)** | 1  1.7 (1.27,2.26)** |
| Depression  No  Mild  Moderate  Severe | 1  1.04 (0.74,1.48)  0.71 (0.4,1.27)  13.43 (2.26,79.89)** | 1  1.02 (0.72,1.45)  0.70 (0.39,1.25)  12.31 (2.07,73.33)** |
| Anxiety  No  Mild  Moderate  Severe | 1  1.08 (0.81,1.46)  0.86 (0.57,1.28)  0.66 (0.28,1.57) | 1  1.08 (0.8,1.45)  0.86 (0.57,1.29)  0.66 (0.28,1.56) |
| AH4 score (literacy/numeracy) | 0.97 (0.96,0.99)** | 0.97 (0.96,0.99)** |
| Rosenburg self-esteem score | 0.98 (0.94,1.02) | 0.98 (0.94,1.02) |
| Number community clubs involved in  None  One  Two  Three  Four or more | 1  1.02 (0.78,1.33)  0.78 (0.56,1.09)  1.06 (0.71,1.58)  0.96 (0.56,1.62)) | 1  1.02 (0.78,1.33)  0.79 (0.57,1.1)  1.04 (0.7,1.56)  0.94 (0.55,1.59) |
| Not limited by LTC  Limiting longstanding illness | 1  1.07 (0.7,1.63)  1.28 (0.87,1.89) |  |
| Maximum Educational Achievement by age 35  No Qualifications  Standard Grades  Apprenticeship  Higher  HND  Degree | 1  1.51 (1.03,2.2)**  0.96 (0.68,1.34)  1.2 (0.74,1.95)  2.65 (1.36,5.16)**  1.52 (0.98,2.36) | 1  1.5 (1.02,2.19)**  0.95 (0.68,1.33)  1.2 (0.74,1.94)  2.59 (1.33,5.04)**  1.5 (0.96,2.33) |
| Health Locus of Control statement | Health Locus of Control statements |  |
| I have the power to make myself well  Agree Strongly  Agree quite a bit  Agree a little  Disagree a little  Disagree quite a bit  Disagree a lot | 1  1.1 (0.75,1.63)  0.68 (0.45,1.02)  0.54 (0.33,0.9)  0.68 (0.4,1.17)  0.53 (0.3,0.95) | 1  1.11 (0.75,1.65)  0.69 (0.46,1.04)  0.54 (0.33,0.9)  0.7 (0.41,1.2)  0.52 (0.29,0.93) |
| I have no control over being ill  Agree Strongly  Agree quite a bit  Agree a little  Disagree a little  Disagree quite a bit  Disagree a lot | 1  1.02 (0.59,1.77)  1.25 (0.74,2.14)  1.63 (0.94,2.83)  1.26 (0.74,2.14)  1.32 (0.81,2.16) | 1  0.99 (0.57,1.71)  1.24 (0.72,2.11)  1.58 (0.91,2.74)  1.22 (0.72,2.08)  1.32 (0.81,2.16) |
| Regular doctor visits reduce health  Agree Strongly  Agree quite a bit  Agree a little  Disagree a little  Disagree quite a bit  Disagree a lot | 1  1.77 (1.07,2.95)  1.08 (0.69,1.69)  0.83 (0.48,1.41)  0.98 (0.6,1.62)  0.88 (0.55,1.4) | 1  1.77 (1.07,2.95)  1.08 (0.69,1.69)  0.79 (0.46,1.36)  0.95 (0.58,1.56)  0.85 (0.53,1.36) |
| Accidental happening influence health  Agree Strongly  Agree quite a bit  Agree a little  Disagree a little  Disagree quite a bit  Disagree a lot | 1  1.48 (0.78,2.82)  1.12 (0.62,2.02)  1.39 (0.75,2.58)  1.26 (0.68,2.33)  1 (0.56,1.8) | 1  1.48 (0.78,2.81)  1.12 (0.62,2.03)  1.4 (0.75,2.59)  1.3 (0.7,2.4)  1.02 (0.57,1.83) |
| Only doctors can maintain health  Agree Strongly  Agree quite a bit  Agree a little  Disagree a little  Disagree quite a bit  Disagree a lot | 1  1.31 (0.71,2.43)  0.6 (0.33,1.11)  0.74 (0.39,1.38)  0.91 (0.49,1.68)  1.03 (0.57,1.84) | 1  1.33 (0.71,2.46)  0.6 (0.32,1.11)  0.73 (0.39,1.38)  0.91 (0.49,1.69)  1.03 (0.57,1.85) |
| I am responsible for my health  Agree Strongly  Agree quite a bit  Agree a little  Disagree a little  Disagree quite a bit  Disagree a lot | 1  1.09 (0.79,1.51)  1.69 (1.14,2.51)*  1.59 (0.96,2.63)  1.24 (0.62,2.46)  0.65 (0.31,1.36) | 1  1.09 (0.79,1.51)  1.67 (1.13,2.49)*  1.53 (0.92,2.55)  1.25 (0.63,2.48)  0.69 (0.33,1.45) |
| Others are responsible for my health  Agree Strongly  Agree quite a bit  Agree a little  Disagree a little  Disagree quite a bit  Disagree a lot | 1  1.25 (0.7,2.24)  0.85 (0.49,1.49)  1.65 (0.92,2.94)  0.76 (0.44,1.3)  1.15 (0.69,1.93) | 1  1.27 (0.71,2.26)  0.88 (0.5,1.54)  1.67 (0.93,2.99)  0.77 (0.44,1.32)  1.19 (0.71,1.99) |
| It’s my fault when things go wrong with my health  Agree Strongly  Agree quite a bit  Agree a little  Disagree a little  Disagree quite a bit  Disagree a lot | 1  1.25 (0.76,2.05)  1.35 (0.83,2.17)  1.33 (0.81,2.17)  1.46 (0.88,2.42)  1.16 (0.7,1.9) | 1  1.23 (0.75,2.02)  1.37 (0.85,2.21)  1.34 (0.82,2.2)  1.47 (0.88,2.43)  1.16 (0.7,1.9) |
| When I am ill, I let nature run its course  Agree Strongly  Agree quite a bit  Agree a little  Disagree a little  Disagree quite a bit  Disagree a lot | 1  1.08 (0.67,1.74)  1.05 (0.67,1.64)  1.13 (0.68,1.88)  1.06 (0.66,1.72)  1.06 (0.66,1.68) | 1  1.06 (0.66,1.71)  1.03 (0.66,1.6)  1.14 (0.69,1.89)  1.04 (0.64,1.68)  1.04 (0.65,1.65) |
| When I’m health it’s because I am lucky  Agree Strongly  Agree quite a bit  Agree a little  Disagree a little  Disagree quite a bit  Disagree a lot | 1  0.8 (0.47,1.37)  0.93 (0.54,1.6)  0.85 (0.49,1.49)  0.92 (0.51,1.65)  1.04 (0.58,1.86) | 1  0.79 (0.46,1.35)  0.94 (0.55,1.61)  0.86 (0.49,1.5)  0.93 (0.52,1.68)  1.07 (0.59,1.92) |
| Wellbeing depends on taking care of yourself  Agree Strongly  Agree quite a bit  Agree a little  Disagree a little  Disagree quite a bit  Disagree a lot | 1  1.02 (0.65,1.6)  1.08 (0.69,1.67)  1.14 (0.69,1.87)  1.18 (0.73,1.92)  0.77 (0.47,1.26) | 1  1.01 (0.65,1.59)  1.09 (0.7,1.69)  1.16 (0.7,1.91)  1.17 (0.72,1.91)  0.76 (0.46,1.25) |
| Illness means you have not cared for yourself  Agree Strongly  Agree quite a bit  Agree a little  Disagree a little  Disagree quite a bit  Disagree a lot | 1  1.04 (0.76,1.43)  1.23 (0.81,1.88)  1.76 (0.86,3.59)  0.64 (0.25,1.68)  1.96 (0.82,4.69) | 1  1.04 (0.76,1.42)  1.22 (0.8,1.86)  1.68 (0.82,3.42)  0.64 (0.25,1.67)  1.95 (0.81,4.68) |
| Care from others helps me to get well  Agree Strongly  Agree quite a bit  Agree a little  Disagree a little  Disagree quite a bit  Disagree a lot | 1  0.48 (0.32,0.72)**  0.62 (0.41,0.94)**  0.4 (0.25,0.65)**  0.53 (0.33,0.85)**  0.77 (0.46,1.27) | 1  0.48 (0.32,0.73)**  0.62 (0.41,0.95)**  0.41 (0.25,0.66)**  0.53 (0.33,0.86)**  0.76 (0.46,1.26) |
| Illness is luck  Agree Strongly  Agree quite a bit  Agree a little  Disagree a little  Disagree quite a bit  Disagree a lot | 1  0.87 (0.58,1.29)  1.1 (0.73,1.64)  1.45 (0.89,2.36)  0.98 (0.56,1.7)  0.93 (0.51,1.67) | 1  0.87 (0.59,1.29)  1.1 (0.74,1.64)  1.44 (0.88,2.35)  0.99 (0.57,1.73)  0.93 (0.51,1.67) |
| Looking after myself keeps me healthy  Agree Strongly  Agree quite a bit  Agree a little  Disagree a little  Disagree quite a bit  Disagree a lot | 1  0.99 (0.63,1.56)  1.24 (0.8,1.94)  0.82 (0.49,1.35)  1.12 (0.7,1.81)  1.55 (0.93,2.56) | 1  1.02 (0.65,1.61)  1.26 (0.8,1.97)  0.84 (0.51,1.39)  1.15 (0.72,1.85)  1.59 (0.96,2.63) |
| Doctor’s orders keep me healthy  Agree Strongly  Agree quite a bit  Agree a little  Disagree a little  Disagree quite a bit  Disagree a lot | 1  0.69 (0.37,1.32)  0.72 (0.4,1.31)  0.58 (0.31,1.06)  0.62 (0.33,1.15)  0.89 (0.49,1.61) | 1  0.72 (0.38,1.36)  0.74 (0.41,1.33)  0.59 (0.32,1.08)  0.63 (0.34,1.17)  0.91 (0.5,1.66) |
| I can usually stay healthy by taking good care of myself  Agree Strongly  Agree quite a bit  Agree a little  Disagree a little  Disagree quite a bit  Disagree Strongly | 1  1.23 (0.86,1.76)  1.2 (0.79,1.83)  1.24 (0.71,2.16)  1.57 (0.8,3.07)  0.98 (0.38,2.56) | 1  1.24 (0.86,1.77)  1.22 (0.8,1.85)  1.29 (0.74,2.24)  1.59 (0.81,3.12)  0.95 (0.36,2.46) |
| Following the doctors order to the letter is the way to stay healthy  Agree Strongly  Agree quite a bit  Agree a little  Disagree a little  Disagree quite a bit  Disagree Strongly | 1  0.8 (0.56,1.13)  1.01 (0.7,1.47)  0.57 (0.34,0.97)  1.14 (0.67,1.95)  0.53 (0.28,1) | 1  0.79 (0.56,1.12)  1.01 (0.7,1.47)  0.57 (0.34,0.96)  1.13 (0.66,1.93)  0.53 (0.28,1.01) |
| I have little control over what happens to me  Strongly agree  Agree  Disagree  Strongly disagree | 1  0.51 (0.27,0.98)  0.47 (0.25,0.9)  0.57 (0.28,1.14) | 1  0.51 (0.27,0.98)  0.47 (0.25,0.9)  0.55 (0.27,1.11) |
| There is no way I can solve some of the problems I have  Strongly agree  Agree  Disagree  Strongly disagree | 1  0.91 (0.47,1.79)  0.86 (0.43,1.74)  0.83 (0.38,1.82) | 1  0.93 (0.47,1.83)  0.86 (0.43,1.75)  0.85 (0.39,1.86) |
| There is little I can do to change many of the important things in my life  Strongly agree  Agree  Disagree  Strongly disagree | 1  0.61 (0.29,1.27)  0.56 (0.26,1.19)  0.49 (0.21,1.16) | 1  0.6 (0.29,1.25)  0.54 (0.25,1.16)  0.47 (0.2,1.12) |
| Sometimes I feel helpless dealing with the problems in life  Strongly agree  Agree  Disagree  Strongly disagree | 1  1.13 (0.54,2.37)  1.22 (0.56,2.65)  1.08 (0.47,2.47) | 1  1.12 (0.54,2.36)  1.22 (0.56,2.65)  1.08 (0.47,2.48) |
| Sometimes I feel I am pushed around in life  Strongly agree  Agree  Disagree  Strongly disagree | 1  0.88 (0.4,1.94)  1.38 (0.63,3.04)  1.19 (0.51,2.79) | 1  0.89 (0.41,1.94)  1.42 (0.65,3.11)  1.21 (0.52,2.84) |
| What happens in the future depends mostly on me  Strongly agree  Agree  Disagree  Strongly disagree | 1  1 (0.67,1.49)  1.16 (0.72,1.88)  0.55 (0.27,1.11) | 1  1.01 (0.68,1.51)  1.16 (0.72,1.88)  0.55 (0.27,1.11) |
| I can do just about anything I set my mind to  Strongly agree  Agree  Disagree  Strongly disagree | 1  1 (0.65,1.53)  0.82 (0.49,1.37)  1.28 (0.59,2.76) | 1  1 (0.65,1.54)  0.83 (0.5,1.39)  1.27 (0.59,2.74) |

**Table S8: Table demonstrating Odds Ratio for self-reported hospital admission for Expressing Capacity Variables when not adjusting (Model 1) or adjusting (Model 2) for presence of absence of multimorbidity.**

| Variables | Odds Ratio for self-reported hospital admission in the last year (95% Confidence Interval) | |
| --- | --- | --- |
|  | Model 1 | Model 2 |
| Over the last 12 months would you say your health on the whole has been……  Good  Fairly good  Not good | 1  2.82 (1.94,4.1)**  7.92 (4.64,13.54)** | 1  2.71 (1.86,3.93)**  7.39 (4.33,12.66)** |
| Would you say that for someone your own age your health in general is…  Excellent  Good  Fair  Poor | 1  0.78 (0.51,1.2)  0.66 (0.38,1.16)  0.78 (0.37,1.64) | 1  0.76 (0.5,1.17)  0.64 (0.36,1.11)  0.75 (0.35,1.58) |
| Registered disability  No  Yes | 1  2.00 (1.34,2.97)** | 1  1.97 (1.32,2.93)** |
| Depression  No  Mild  Moderate  Severe | 1  1.1 (0.71,1.68)  1.32 (0.69,2.51)  2.47 (0.07,51.52) | 1  1.08 (0.7,1.66)  1.32 (0.68,2.5)  2.31 (0.07,45.81) |
| Anxiety  No  Mild  Moderate  Severe | 1  1.00 (0.7,1.41)  0.79 (0.5,1.23)  1.08 (0.49,2.3) | 1  0.98 (0.69,1.38)  0.78 (0.49,1.22)  1.01 (0.46,2.15) |
| AH4 score (literacy/numeracy) | 0.98 (0.97,1) | 0.98 (0.97,1) |
| Rosenburg self-esteem score | 1 (0.96,1.05) | 1 (0.96,1.05) |
| Number community clubs involved in  None  One  Two  Three  Four or more | 1  0.95 (0.69,1.32)  1.46 (1,2.11)*  0.87 (0.5,1.44)  1.45 (0.81,2.53) | 1  0.94 (0.68,1.3)  1.47 (1.01,2.13)*  0.84 (0.49,1.41)  1.45 (0.81,2.53) |
| Not limited by LTC  Limiting longstanding illness | 1  1.1 (0.82,1.48) | 1  1 (0.73,1.35) |
| Maximum Educational Achievement by age 35  No Qualifications  Standard Grades  Apprenticeship  Higher  HND  Degree | 1  1.36 (0.89,2.05)  1.35 (0.89,2.04)  1.08 (0.6,1.89)  1.68 (0.76,3.52)  0.94 (0.56,1.58) | 1  1.33 (0.87,2.01)  1.34 (0.88,2.02)  1.05 (0.58,1.84)  1.58 (0.71,3.32)  0.89 (0.52,1.5) |
| Health Locus of Control statement |  |  |
| I have the power to make myself well  Agree Strongly  Agree quite a bit  Agree a little  Disagree a little  Disagree quite a bit  Disagree a lot | 1  0.97 (0.61,1.57)  0.75 (0.46,1.22)  0.97 (0.55,1.73)  0.82 (0.42,1.59)  0.75 (0.37,1.49) | 1  0.99 (0.62,1.6)  0.77 (0.47,1.26)  0.97 (0.55,1.73)  0.83 (0.42,1.61)  0.76 (0.37,1.5) |
| I have no control over being ill  Agree Strongly  Agree quite a bit  Agree a little  Disagree a little  Disagree quite a bit  Disagree a lot | 1  0.87 (0.45,1.68)  0.78 (0.41,1.5)  1.07 (0.55,2.09)  0.49 (0.25,0.95)*  0.75 (0.41,1.39) | 1  0.85 (0.44,1.66)  0.77 (0.41,1.49)  1.06 (0.55,2.07)  0.48 (0.25,0.94)*  0.75 (0.41,1.39) |
| Regular doctor visits reduce health  Agree Strongly  Agree quite a bit  Agree a little  Disagree a little  Disagree quite a bit  Disagree a lot | 1  1.74 (0.88,3.5)  1.57 (0.83,3.06)  0.93 (0.46,1.93)  1.17 (0.59,2.35)  1.28 (0.67,2.5) | 1  1.7 (0.86,3.41)  1.56 (0.82,3.03)  0.92 (0.45,1.9)  1.16 (0.59,2.34)  1.29 (0.67,2.51) |
| Accidental happening influence health  Agree Strongly  Agree quite a bit  Agree a little  Disagree a little  Disagree quite a bit  Disagree a lot | 1  1.35 (0.63,2.99)  0.93 (0.46,1.95)  1.21 (0.58,2.57)  1.01 (0.49,2.16)  1.7 (0.86,3.51) | 1  1.33 (0.62,2.94)  0.91 (0.45,1.91)  1.18 (0.57,2.51)  1.01 (0.49,2.15)  1.68 (0.84,3.47) |
| Only doctors can maintain health  Agree Strongly  Agree quite a bit  Agree a little  Disagree a little  Disagree quite a bit  Disagree a lot | 1  1.3 (0.57,2.98)  0.96 (0.43,2.17)  1.9 (0.88,4.19)  1.93 (0.91,4.18)  1.72 (0.83,3.66) | 1  1.32 (0.58,3.05)  0.95 (0.42,2.16)  1.9 (0.88,4.19)  1.91 (0.9,4.16)  1.66 (0.8,3.54) |
| I am responsible for my health  Agree Strongly  Agree quite a bit  Agree a little  Disagree a little  Disagree quite a bit  Disagree a lot | 1  1.08 (0.74,1.58)  1.25 (0.79,1.98)  0.5 (0.24,0.98)  1.41 (0.64,2.97)  0.77 (0.31,1.8) | 1  1.09 (0.75,1.6)  1.25 (0.79,1.97)  0.48 (0.23,0.95)  1.39 (0.63,2.95)  0.79 (0.32,1.86) |
| Others are responsible for my health  Agree Strongly  Agree quite a bit  Agree a little  Disagree a little  Disagree quite a bit  Disagree a lot | 1  1.8 (0.85,3.89)  1.72 (0.85,3.6)  1.97 (0.94,4.24)  1.54 (0.76,3.23)  1.7 (0.88,3.4) | 1  1.78 (0.84,3.85)  1.75 (0.86,3.67)  1.97 (0.94,4.23)  1.58 (0.78,3.3)  1.75 (0.91,3.5) |
| It’s my fault when things go wrong with my health  Agree Strongly  Agree quite a bit  Agree a little  Disagree a little  Disagree quite a bit  Disagree a lot | 1  0.74 (0.4,1.37)  0.89 (0.49,1.61)  0.75 (0.4,1.39)  1.23 (0.68,2.24)  0.85 (0.46,1.57) | 1  0.71 (0.38,1.33)  0.87 (0.48,1.58)  0.73 (0.39,1.36)  1.21 (0.67,2.21)  0.83 (0.45,1.52) |
| When I am ill, I let nature run its course  Agree Strongly  Agree quite a bit  Agree a little  Disagree a little  Disagree quite a bit  Disagree a lot | 1  0.68 (0.38,1.23)  1.09 (0.64,1.89)  1.15 (0.63,2.1)  0.95 (0.53,1.69)  1.08 (0.61,1.91) | 1  0.67 (0.37,1.21)  1.07 (0.63,1.86)  1.15 (0.63,2.1)  0.94 (0.53,1.68)  1.07 (0.61,1.89) |
| When I’m health it’s because I am lucky  Agree Strongly  Agree quite a bit  Agree a little  Disagree a little  Disagree quite a bit  Disagree a lot | 1  0.71 (0.34,1.46)  0.67 (0.33,1.38)  0.51 (0.25,1.08)  0.59 (0.29,1.24)  0.63 (0.3,1.33) | 1  0.7 (0.34,1.45)  0.68 (0.34,1.39)  0.52 (0.25,1.1)  0.6 (0.29,1.26)  0.65 (0.31,1.37) |
| Wellbeing depends on taking care of yourself  Agree Strongly  Agree quite a bit  Agree a little  Disagree a little  Disagree quite a bit  Disagree a lot | 1  1.21 (0.68,2.16)  0.93 (0.53,1.67)  1.16 (0.62,2.19)  0.96 (0.52,1.78)  1.06 (0.57,1.96) | 1  1.21 (0.68,2.18)  0.94 (0.53,1.68)  1.16 (0.61,2.2)  0.93 (0.51,1.74)  1.05 (0.57,1.95) |
| Illness means you have not cared for yourself  Agree Strongly  Agree quite a bit  Agree a little  Disagree a little  Disagree quite a bit  Disagree a lot | 1  1.05 (0.73,1.51)  1.42 (0.86,2.32)  1.03 (0.37,2.61)  0.64 (0.2,1.81)  1.37 (0.35,4.41) | 1  1.05 (0.73,1.51)  1.42 (0.86,2.33)  1.02 (0.37,2.56)  0.63 (0.2,1.81)  1.37 (0.35,4.43) |
| Care from others helps me to get well  Agree Strongly  Agree quite a bit  Agree a little  Disagree a little  Disagree quite a bit  Disagree a lot | 1  0.68 (0.4,1.16)  1.09 (0.65,1.85)  0.82 (0.47,1.43)  0.78 (0.43,1.41)  0.98 (0.53,1.82) | 1  0.69 (0.41,1.19)  1.12 (0.66,1.89)  0.85 (0.49,1.5)  0.82 (0.45,1.48)  1.01 (0.54,1.87) |
| Illness is luck  Agree Strongly  Agree quite a bit  Agree a little  Disagree a little  Disagree quite a bit  Disagree a lot | 1  1.08 (0.66,1.78)  0.8 (0.49,1.34)  0.73 (0.4,1.34)  0.97 (0.51,1.84)  1.07 (0.52,2.18) | 1  1.07 (0.65,1.76)  0.79 (0.48,1.33)  0.72 (0.39,1.32)  0.95 (0.49,1.81)  1.04 (0.5,2.12) |
| Looking after myself keeps me healthy  Agree Strongly  Agree quite a bit  Agree a little  Disagree a little  Disagree quite a bit  Disagree a lot | 1  0.9 (0.53,1.54)  1.12 (0.65,1.94)  0.84 (0.46,1.52)  1.03 (0.59,1.83)  0.86 (0.46,1.59) | 1  0.92 (0.54,1.59)  1.13 (0.65,1.96)  0.87 (0.48,1.59)  1.09 (0.61,1.94)  0.88 (0.47,1.63) |
| Doctor’s orders keep me healthy  Agree Strongly  Agree quite a bit  Agree a little  Disagree a little  Disagree quite a bit  Disagree a lot | 1  0.69 (0.31,1.55)  0.83 (0.4,1.76)  0.96 (0.46,2.08)  0.87 (0.41,1.87)  0.85 (0.41,1.82) | 1  0.7 (0.32,1.59)  0.84 (0.41,1.8)  0.95 (0.45,2.06)  0.86 (0.41,1.87)  0.87 (0.42,1.86) |
| I can usually stay healthy by taking good care of myself  Agree Strongly  Agree quite a bit  Agree a little  Disagree a little  Disagree quite a bit  Disagree Strongly | 1  1.11 (0.73,1.68)  0.88 (0.53,1.45)  0.95 (0.48,1.87)  0.88 (0.37,2.01)  0.51 (0.13,1.75) | 1  1.1 (0.73,1.67)  0.88 (0.53,1.47)  0.95 (0.47,1.87)  0.85 (0.35,1.96)  0.48 (0.12,1.67) |
| Following the doctors order to the letter is the way to stay healthy  Agree Strongly  Agree quite a bit  Agree a little  Disagree a little  Disagree quite a bit  Disagree Strongly | 1  0.99 (0.65,1.51)  0.79 (0.5,1.25)  1.04 (0.59,1.81)  0.78 (0.41,1.45)  0.54 (0.24,1.17) | 1  0.98 (0.64,1.49)  0.79 (0.5,1.25)  1.01 (0.57,1.76)  0.79 (0.41,1.47)  0.53 (0.24,1.15) |
| I have little control over what happens to me  Strongly agree  Agree  Disagree  Strongly disagree | 1  1.96 (0.77,5.39)  2 (0.79,5.53)  1.4 (0.53,4.08) | 1  2 (0.79,5.49)  2.05 (0.81,5.67)  1.41 (0.53,4.1) |
| There is no way I can solve some of the problems I have  Strongly agree  Agree  Disagree  Strongly disagree | 1  0.84 (0.37,1.98)  0.63 (0.27,1.52)  0.54 (0.21,1.45) | 1  0.85 (0.37,2.01)  0.63 (0.27,1.54)  0.55 (0.21,1.46) |
| There is little I can do to change many of the important things in my life  Strongly agree  Agree  Disagree  Strongly disagree | 1  0.51 (0.21,1.28)  0.57 (0.23,1.48)  0.58 (0.21,1.66) | 1  0.55 (0.23,1.39)  0.6 (0.24,1.58)  0.61 (0.22,1.76) |
| Sometimes I feel helpless dealing with the problems in life  Strongly agree  Agree  Disagree  Strongly disagree | 1  2.39 (0.88,7)  3.17 (1.13,9.64)**  3.36 (1.12,10.9)** | 1  2.25 (0.83,6.57)  3.03 (1.09,9.19)**  3.17 (1.06,10.22)** |
| Sometimes I feel I am pushed around in life  Strongly agree  Agree  Disagree  Strongly disagree | 1  0.67 (0.29,1.66)  0.63 (0.27,1.56)  0.7 (0.27,1.87) | 1  0.7 (0.3,1.76)  0.67 (0.29,1.67)  0.75 (0.29,2.02) |
| What happens in the future depends mostly on me  Strongly agree  Agree  Disagree  Strongly disagree | 1  0.67 (0.44,1.05)  0.47 (0.27,0.81)  0.66 (0.28,1.49) | 1  0.66 (0.42,1.03)  0.46 (0.26,0.79)  0.64 (0.27,1.45) |
| I can do just about anything I set my mind to  Strongly agree  Agree  Disagree  Strongly disagree | 1  0.87 (0.53,1.42)  0.95 (0.53,1.73)  1.36 (0.52,3.47) | 1  0.88 (0.54,1.45)  0.96 (0.53,1.75)  1.36 (0.52,3.47) |

**Table S9: demonstrating Hazard Ratio for Community Capacity Variables when not adjusting (Model 1) or adjusting (Model 2) for presence of absence of multimorbidity.**

| Variables | Hazard ratio for mortality  (95% Confidence interval). | |
| --- | --- | --- |
|  | Model 1 | Model 2 |
| Exchange Small Favours with those who live nearby  Yes  No | 1  1.03 (0.88,1.21) | 1  1.02 (0.87,1.2) |
| How do you feel about the area you live in (faces scale)  1 Most Positive  2  3  4  5  6  7 Most Negative | 1  0.86 (0.73,1.02)  0.91 (0.75,1.1)  1.03 (0.78,1.35)  0.76 (0.48,1.21)  1.18 (0.72,1.92)  1.58 (1.04,2.39) | 1  0.84 (0.71,0.99)*  0.9 (0.74,1.09)  0.98 (0.75,1.29)  0.69 (0.44,1.09)  1.13 (0.69,1.83)  1.47 (0.97,2.23) |
| How do you feel about walking around the area after dark? Would you say that you  Never  Try to avoid  Feel uncomfortable  Have no worries | 1  0.7 (0.57,0.87)  0.66 (0.52,0.84)  0.63 (0.53,0.76) | 1  0.73 (0.59,0.9)  0.7 (0.55,0.89)  0.65 (0.54,0.78) |
| Around the area you live would you say vandalism is a problem?  Serious Problem  Minor Problem  No Problem | 1  1.03 (0.82,1.3)  1.01 (0.78,1.3) | 1  0.98 (0.78,1.23)  0.97 (0.75,1.24) |
| Around the area you live would you say litter is a problem?  Serious Problem  Minor Problem  No Problem | 1  1.01 (0.83,1.24)  1.12 (0.9,1.39) | 1  1.01 (0.83,1.23)  1.11 (0.9,1.38) |
| Around the area you live would you say assaults are a problem?  Serious Problem  Minor Problem  No Problem | 1  0.98 (0.74,1.29)  0.92 (0.7,1.21) | 1  0.98 (0.74,1.29)  0.91 (0.69,1.2) |
| Around the area you live would you say burglaries are a problem?  Serious Problem  Minor Problem  No Problem | 1  0.93 (0.75,1.16)  1.05 (0.84,1.31) | 1  0.96 (0.77,1.19)  1.06 (0.85,1.33) |
| Around the area you live would you say young people causing disturbances are a problem?  Serious Problem  Minor Problem  No Problem | 1  1.01 (0.77,1.32)  0.98 (0.75,1.29) | 1  1.05 (0.8,1.38)  1.03 (0.78,1.35) |

**Table S10 demonstrating Odds Ratio for self-reported hospital admission for Mobilising Capacity Variables when not adjusting (Model 1) or adjusting (Model 2) for presence of absence of multimorbidity.**

| Variables | Odds Ratio for self-reported hospital admission in the last year (95% Confidence Interval) | |
| --- | --- | --- |
|  | Model 1 | Model 2 |
| Exchange Small Favours with those who live nearby  Yes  No | 1  0.96 (0.79,1.16) | 1  0.96 (0.79,1.16) |
| How do you feel about the area you live in (faces scale)  1 Most Positive  2  3  4  5  6  7 Most Negative | 1  1.17 (0.95,1.43)  1.25 (0.99,1.58)  1.01 (0.7,1.44)  1.00 (0.58,1.65)  1.96 (1.09,3.39)*  1.18 (0.64,2.08) | 1  1.15 (0.94,1.42)  1.24 (0.98,1.57)  0.97 (0.67,1.38)  0.93 (0.53,1.53)  1.86 (1.03,3.23)*  1.14 (0.62,2.02) |
| How do you feel about walking around the area after dark? Would you say that you  Never  Try to avoid  Feel uncomfortable  Have no worries | 1  0.80 (0.6,1.05)  0.61 (0.45,0.83)**  0.71 (0.55,0.91)** | 1  0.84 (0.64,1.11)  0.64 (0.47,0.87)**  0.75 (0.59,0.96)* |
| Around the area you live would you say vandalism is a problem?  Serious Problem  Minor Problem  No Problem | 1  0.96 (0.73,1.27)  0.99 (0.73,1.35) | 1  0.95 (0.72,1.26)  0.98 (0.72,1.34) |
| Around the area you live would you say litter is a problem?  Serious Problem  Minor Problem  No Problem | 1  1.01 (0.79,1.3)  1.17 (0.9,1.53) | 1  0.98 (0.77,1.26)  1.13 (0.87,1.48) |
| Around the area you live would you say assaults are a problem?  Serious Problem  Minor Problem  No Problem | 1  0.63 (0.45,0.87)**  0.63 (0.45,0.87)** | 1  0.66 (0.47,0.92)**  0.67 (0.48,0.93)** |
| Around the area you live would you say burglaries are a problem?  Serious Problem  Minor Problem  No Problem | 1  1.14 (0.9,1.45)  1.26 (0.98,1.62) | 1  1.10 (0.86,1.4)  1.20 (0.94,1.55) |
| Around the area you live would you say young people causing disturbances are a problem?  Serious Problem  Minor Problem  No Problem | 1  1.20 (0.86,1.69)  1.02 (0.73,1.44) | 1  1.21 (0.86,1.71)  1.05 (0.75,1.48) |

1. Rosenburg M: **Society and the adolescent self Image**. Princeton New Jersey: University Press; 1965.

1. *Green nodes represent potential explanatory variables, dark grey dots are potential confounders. Light grey dots are important unmeasured capacity factors. As the known relationships between the variables are imputed the software identifies causal pathways (green), and bias pathways (pink) guiding what variables should be identified as confounders and adjusted for in the model.* [↑](#footnote-ref-1)
